# A comparison of genome cohort participants’ genetic knowledge and preferences to receive genetic results before and after a genetics workshop

**DOI:** 10.1038/s10038-018-0494-z

**Published:** 2018-09-05

**Authors:** Kayono Yamamoto, Atsushi Shimizu, Fumie Aizawa, Hiroshi Kawame, Tomoharu Tokutomi, Akimune Fukushima

**Affiliations:** 1 Division of Innovation and Education, Iwate Tohoku Medical Megabank Organization, Disaster Reconstruction Center, Iwate Medical University, Iwate, Japan; 2 Department of Clinical Genetics, School of Medicine, Iwate Medical University, Iwate, Japan; 3 Division of Biomedical Information Analysis, Iwate Tohoku Medical Megabank Organization, Disaster Reconstruction Center, Iwate Medical University, Iwate, Japan; 4 Center for Liberal Arts and Sciences, Iwate Medical University, Iwate, Japan; 5 Department of Education and Training, Tohoku Medical Megabank Organization, Tohoku University, Sendai, Japan

**Keywords:** Patient education, Health sciences

## Abstract

Several biobanks have begun returning genetic results to individuals, making the development of public genetic literacy an urgent task for their effective use. No research exists regarding the effects of genetic education on biobank participants, so we conducted genetics workshops with specialists, and surveyed differences in the participants’ (*n* = 112) preferences to receive their own genetic information by disease categories and their genetic knowledge using questionnaires before and after the workshops. Almost 90% of our participants were over 60 years old, which was similar to our previous preference research. The preference to receive five of the six categories of genetic information (lifestyle diseases, pharmacogenetics, adult-onset non-clinically actionable diseases, non-clinically actionable multifactorial diseases, and all genetic information) was slightly but significantly decreased after the genetics workshop. More participants preferred to receive genetic results regarding lifestyle diseases, pharmacogenetics, and adult-onset clinically actionable diseases after the workshop, while less participants preferred to receive information regarding adult-onset non-clinically actionable diseases, non-clinically actionable multifactorial diseases, and all genetic information. Total genetic knowledge scores significantly increased after the workshop (before: 11.89, after: 13.30, *p* < 0.001). Our findings suggest that genetics workshops are useful to improve the genetic literacy of genome cohort participants.

## Introduction

The technological development of next-generation sequencing and bioinformatics has resulted in whole-genome/exome sequencing being conducted in clinical settings [1]. Large-scale genomic screens have attempted to biobank DNA samples and identify various gene mutations that cause clinically actionable genetic diseases [2]. Several biobanks have begun returning results to individuals. The Coriell Personalized Medicine Collaborative, which is based on both disease-specific and healthy cohort studies, has begun reporting the relative risk for common complex diseases to the participants [3]. A few population-based biobank studies on single-gene diseases have also provided subjects with their genetic results. Haukkala et al. reported on the returning of genetic results regarding heritable long QT syndrome in a Finnish population-based cohort study in which 65% (*n* = 17) of subjects positive for long QT syndrome, as detected by genotyping, participated in a follow-up study. Many participants were surprised by the positive result, but deemed the information useful for their health [4]. The Tohoku Medical Megabank Project, a population-based biobank in Japan [5], has begun returning individual genetic information regarding familial hypercholesterolemia. We have previously studied the population-based biobank participants’ preferences to receive genetic test results, revealing that nearly 90% of participants enrolled in the Tohoku Medical Megabank Project cohort study expressed a preference to receive their results [6]. However, participant preferences based on disease categories have not been systematically studied.

During the last decade, human genetics technology has dramatically progressed and genetics-centered health-care is becoming a reality. Therefore, developing public genetic literacy is urgently required to help people understand their genetic information and use it rationally and effectively [7]. Appropriate science education programs have been developed in schools [8, 9] and several genetic education programs are being offered in schools and communities [10]. Intervention studies have also examined the effects of genetic lectures conducted by primary care providers [11]. However, there is no research regarding the effects of genetic education on biobank participants. Therefore, we surveyed the differences among participant preferences to receive their own genetic information by disease categories, and examined the effects of participation in a basic genetics workshop run by genetic specialists who usually offer genetic counseling in clinical situations on the participants’ genetic knowledge, and preferences to receive genetic results.

## Materials and methods

### Study population

The study population was recruited from participants who attended health checks at Iwate Tohoku Medical Megabank satellites (October 2014 to February 2015). We distributed a research participant recruitment leaflet to health check participants, and introduced the details of our research to people who visited our recruitment booth. We conducted an initial face-to-face questionnaire (the before workshop questionnaire) after obtaining informed consent. At a later date, we conducted a workshop providing genetic knowledge to respondents of the first questionnaire, and then repeated the face-to-face questionnaire with the workshop participants (the after-workshop questionnaire).

### Questionnaire development and administration

In this study, we analyzed participants’ responses regarding preferences to receive their genetic test results. We examined differences in the participants’ preferences to receive their own genetic information by disease category, the reasons for the participants’ preferences, and the participants’ preferred method of receiving their genetic information. We also investigated the participants’ genetic knowledge and demographic data.

We developed the questions regarding preferences to receive genetic test results using a literature review, expert consultations, and a pilot study. The detailed process has been previously described [6]. The participants’ preferences regarding the receipt of their genetic information were examined for the following types of diseases: lifestyle diseases (e.g., hypertension, diabetes), pharmacogenetics (e.g., responses to cold remedies, anticancer agents), adult-onset clinically actionable diseases (e.g., hereditary breast and ovarian cancer, hereditary colorectal cancer), adult-onset non-clinically actionable diseases, non-clinically actionable diseases in which the appearance of symptoms is not solely determined by genetics (e.g., Alzheimer’s disease), and all genetic information, regardless of its relation to disease. We did not give an example regarding adult-onset non-clinically actionable diseases because describing specific diseases to be non-clinically actionable in the questionnaire can be offensive to participants affected with the disease. We used a 4-point Likert-type scale, where 1 = “I want to know”, 2 = “If I must answer, then I want to know”, 3 = “If I must answer, then I don’t want to know”, and 4 = “I do not want to know”; we also included a “not sure” choice. The reasons for participants’ preferences to receive their genetic test results were examined using multiple-choice questions. Participants’ preferred method of receiving their genetic information by disease were chosen from the following: from a genetic specialist, from a family doctor, from a regional public health nurse, from a regional pharmacist, by video conference/telephone with a specialist, by mail from genetic testing companies, and on the web page of a genetic testing company. To assess genetic knowledge, we used a Japanese version of the genetic knowledge questionnaire developed by Jallinoja and Aro [12] and have been used in other researches [13, 14], consisting of 16 true or false questions regarding basic and clinical genetics.

### The genetics workshop

The materials used in the genetics workshop were developed by expert consultations between a clinical geneticist, a molecular geneticist, and a certified genetic counselor at Iwate Medical University and Iwate Tohoku Medical Megabank Organization. A clinical geneticist and a molecular geneticist at Tohoku University and Tohoku Medical Megabank Organization revised the draft. The materials also referred to the announcement about revising scientific terms of genetics by The Japan Society of Human Genetics (2009) [15]. The lecture contained basic knowledge regarding genes, DNA, chromosomes, cells, genome, genetic diseases, genetic testing, family trees, and the necessity of carefully handling genetic information. The differences between “genetics” and “hereditary” were specifically explained in the lecture slides, because both words represent the same word, “iden” in Japanese, and therefore, people sometimes confuse the meaning of both. The lecture slides of the genetics workshop are shown as an appendix (Appendix 1-1) and an explanation of the slides is added in Appendix 1-2. The lecture slides can be also viewed at http://iwate-megabank.org/en/genetic/ [16] by clicking the “Comprehensible Explanation of Genetics” button.

The total time of the workshop was almost 60 min. A genetics specialist presented a lecture for about 30 min and addressed participants’ questions, and another genetic specialist and GMRCs (genome medical research coordinator) helped to manage the workshop. The participants were asked to answer the questionnaire after the lecture without referring to the distributed lecture slides. Correct answers were revealed after the participants finished answering the questionnaire.

### Statistical analysis

Statistical analyses of the survey data were performed using IBM SPSS Statistics 23.0. For hypothesis testing, *p*-values < 0.05 were considered statistically significant. Descriptive statistics were used to summarize the demographic data of the participants. Ages were sorted into six categories: (1) 20–29, (2) 30–39, (3) 40–49, (4) 50–59, (5) 60–69, and (6) 70 +. In the genetic knowledge questionnaire, correct answers received 1 point, while wrong answers and unanswered questions received 0 points. Spearman’s rank-order correlation was used to assess the relationships between the genetic knowledge score and the participants’ educational backgrounds, as well as their preferences to receive their genetic information by disease category. To analyze the correlation between the genetic knowledge score and the participants’ educational background levels, the levels were categorized as 1 = junior high school, 2 = high school, 3 = vocational college, junior college, or technical junior college, and 4 = university, undergraduate, or graduate degree. McNemar’s test and the paired *t*-test were used to investigate differences in the participants’ genetic knowledge before and after the genetics workshop. A Kruskal-Wallis H test was used to determine the differences in participants’ preferences to receive their genetic information among the six categories, and pairwise comparisons were performed using Dunn’s test with a Bonferroni correction for multiple comparisons as post hoc analysis. A Wilcoxon signed-rank test was conducted to reveal the participants’ preferences to receive their genetic information by disease category before and after the genetics workshop.

### Ethical statement

Ethical approval was obtained from the institutional review board of Iwate Medical University School of Medicine (approval ID: H26-96). Our study was conducted in accordance with the Declaration of Helsinki, the Japanese Act on the Protection of Personal Information, and the Japan Ethical Guidelines for Medical and Health Research Involving Human Subjects. The questionnaire was accompanied by a participant information sheet on which the participants were asked to write their names and addresses, and to complete and return the questionnaire. Returning the questionnaire implied informed consent.

## Results

### Participant characteristics

We invited 542 people to participate in the study, and conducted a questionnaire-based assessment of the demand for genetic information with 375 participants (a response rate of 70.4%) and their family members in the Iwate Medical University project, at the Yahaba Center and the Kesen Satellite on 28 occasions between October 2014 and February 2015. Among these respondents, 112 participated in genetics workshops, held on 10 occasions at Ofunato Hospital, Yahaba Center, and the Kesen Satellite, and re-answered the questionnaire afterward. The workshop participants’ ages and educational backgrounds are shown in Tables 1 and 2. Almost 90% of our participants were over 60 years old. Of the workshop participants, 51.8% (*n* = 58) were recruited at Yahaba Center and 48.2% (*n* = 54) were recruited at the Kesen Satellite.

**Table 1:** Age distribution of participants (*n* = 112)

Age (years)		Male	Female	Subtotal
20–29	*N*	0	1	1
	(%)	(0.0)	(1.4)	(0.9)
30–39	*N*	0	2	2
	(%)	(0.0)	(2.9)	(1.8)
40–49	*N*	0	2	2
	(%)	(0.0)	(2.9)	(1.8)
50–59	*N*	4	6	10
	(%)	(9.5)	(8.6)	(8.9)
60–69	*N*	16	44	60
	(%)	(38.1)	(62.9)	(53.6)
Over 70	*N*	22	15	37
	(%)	(52.4)	(21.4)	(33.0)
Total	*N*	42	70	112
	(%)	(37.5)	(62.5)	(100)

**Table 2:** Educational background of participants (*n* = 112)

Educational background		Subtotal
Junior high school	*n*	13
	(%)	(11.6)
High school	*n*	57
	(%)	(50.9)
Vocational college	*n*	18
	(%)	(16.1)
Junior college	*n*	7
	(%)	(6.3)
University, undergraduate degree	*n*	15
	(%)	(13.4)
University, graduate degree	*n*	1
	(%)	(0.9)
Other (Technical junior college)	*n*	1
	(%)	(0.9)

### Correlation between genetic knowledge scores and educational backgrounds

Spearman’s rank-order correlation was used to assess the relationship between the participants’ genetic knowledge scores and educational background levels. Before the genetics workshop, there was a positive correlation between the total genetic knowledge score and the participants’ educational background levels (*r*_s_ = 0.239, *p* = 0.011). However, after the genetics workshop, there were no statistically significant correlations between the participants’ total genetic knowledge scores and educational backgrounds (*r*_s_ = 0.077, *p* = 0.417).

### Comparison of participants’ genetic knowledge before and after the genetics workshop

We investigated the differences in the participants’ genetic knowledge before and after the genetics workshop. The correct answer rates of three questions and the total genetic knowledge scores were significantly increased after the workshop, and no questions decreased in score after the workshop. The increased correct answer rates related to the statements “A gene is a disease” (before 78%, after 93%, *p* = 0.001), “Different body parts include different genes” (before 34%, after 77%, *p* < 0.001), and “It has been estimated that a person has 22,000 genes” (before 68%, after 97%, *p* < 0.001). The total genetic knowledge score increased from 11.89 to 13.30 (*p* < 0.001) (Table 3). We also provided a comparison of genetic knowledge between our study group and those in previous studies as an appendix (Appendix 2).

**Table 3:** Comparison of participants’ genetic knowledge before and after the genetics workshop

	Before the workshop (*n* = 112)		After the workshop (*n* = 112)		*p* Value^a^
	(*n*)	(%)	(*n*)	(%)	
1. One can see a gene with the naked eye	100	89	106	95	0.210
2. A gene is a disease	87	78	104	93	0.001**
3. A gene is a molecule that controls hereditary characteristics	81	72	79	71	0.860
4. Genes are inside cells	100	89	105	94	0.267
5. A gene is a piece of DNA	110	98	104	93	0.109
6. A gene is a cell	37	33	42	38	0.486
7. A gene is a part of a chromosome	101	90	103	92	0.804
8. Different body parts include different genes	38	34	86	77	<0.001**
9. Genes are bigger than chromosomes	83	74	93	83	0.112
10. The genotype is not susceptible to human intervention	64	57	70	63	0.417
11. It has been estimated that a person has 22,000 genes	76	68	109	97	<0.001**
12. Healthy parents can have a child with a hereditary disease	94	84	102	91	0.134
13. The onset of certain diseases is due to genes, environment, and lifestyle	94	84	102	91	0.134
14. The carrier of a disease gene may be completely healthy	98	88	105	94	0.118
15. All serious diseases are hereditary	97	87	101	90	0.503
16. The child of a disease gene carrier is always also a carrier of the same disease gene	72	64	79	71	0.381
Overall average score (%)	74.3		83.1		
Overall average score (full score is 16)	11.89		13.30	<0.001**^b^	

Numbers refer to the percentage of participants who answered the question correctly. Before the workshop: our participants’ scores before receiving a basic genetics knowledge workshop; after the workshop: our participants’ scores after receiving a basic genetics knowledge workshop

^a^*p* Values for each question were calculated using McNemar’s test

^b^The overall score *p* Values was calculated using a paired *t*-test

**: *p* < 0.001

### Participants’ preferences to receive their genetic information by disease category

**Fig. 1:**
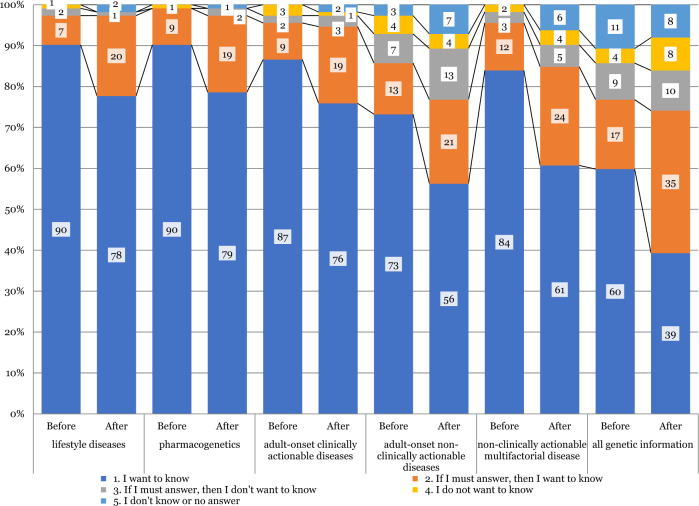
Comparison among participants’ preferences to receive their genetic information by disease category. Pairwise comparisons were performed using Dunn’s procedure with a Bonferroni correction for multiple comparisons. Values are mean ranks unless otherwise stated

The results of descriptive analysis comparing participants’ preferences to receive their genetic information by disease category before and after the genetics workshop are shown in Fig. 1. In the before workshop questionnaire, the score distributions of the participants’ preferences to receive their genetic information seemed dissimilar between disease categories, as assessed by visual inspection of the boxplot, and were significantly different (χ^2^(5) = 32.910, *p* < 0.0001). The results of post hoc analyses are shown in Table 4, which revealed statistically significant differences in participants’ preferences to receive their genetic information between lifestyle diseases and adult-onset non-clinically actionable diseases (303.26 vs. 354.64; adjusted *p* = 0.035), lifestyle diseases and all genetic information (303.26 vs. 380.89; adjusted *p* < 0.001), pharmacogenetics and adult-onset non-clinically actionable diseases (302.38 vs. 354.64; adjusted *p* = 0.029), pharmacogenetics and all genetic information (302.38 vs. 380.89; adjusted *p* < 0.001), adult-onset clinically actionable diseases and all genetic information (315.51 vs. 380.89; adjusted *p* = 0.002), and non-clinically actionable multifactorial diseases and all genetic information (323.58 vs. 380.89; adjusted *p* = 0.014).

**Table 4:** Comparison of the participants’ (*n* = 112) preferences for different disease categories

	Mean rank^a^	Statistically significant differences in participants’ preferences regarding genetic information items (*p* < 0.05)^b^	Mean rank^a^	Statistically significant differences in participants’ preferences regarding genetic information items (*p* < 0.05)^b^
Disease category	Before the genetics workshop		After the genetics workshop	
A: Lifestyle diseases	303.26	A > D, F	280.34	A > D, F
B: Pharmacogenetics	302.38	B > D, F	280.63	B > D, F
C: Adult-onset clinically actionable diseases	315.51	C > F	288.52	C > D, F
D: Adult-onset non-clinically actionable diseases	354.64	D > F D < A, B	351.40	D < A, B, C
E: Non-clinically actionable diseases in which the appearance of symptoms are not solely genetically determined (non-clinically actionable multifactorial diseases)	323.58	E > F	331.70	E > F
F: All genetic information, regardless of its relation to disease (all genetic information)	380.89	F < A, B, C, D, E	497.26	F < A, B, C, E

^a^A smaller mean rank indicates a stronger preference regarding the genetic information

^b^Differences in participants’ preferences for receiving their own genetic information were calculated by Kruskal-Wallis H test

After the workshop, the score distributions of participants’ preferences to receive their genetic information were significantly different between disease categories (χ^2^(5) = 52.927, *p* < 0.001). Post hoc analysis revealed statistically significant differences in the participants’ preferences to receive their genetic information between lifestyle diseases and adult-onset non-clinically actionable diseases (280.34 vs. 351.40; adjusted *p* = 0.01), lifestyle diseases and all genetic information (280.34 vs. 497.26; adjusted *p* < 0.001), pharmacogenetics and adult-onset non-clinically actionable diseases (280.63 vs. 351.40; adjusted *p* = 0.01), pharmacogenetics and all genetic information (280.63 vs. 497.26; adjusted *p* < 0.001), adult-onset clinically actionable diseases and adult-onset non-clinically actionable diseases (288.52 vs. 351.40; adjusted *p* = 0.039), adult-onset clinically actionable diseases and all genetic information (288.52 vs. 497.26; adjusted *p* = 0.002), and non-clinically actionable multifactorial diseases and all genetic information (331.70 vs. 497.26; adjusted *p* = 0.005). Of the six categories, participants’ preferences regarding the genetic information contained in five (lifestyle diseases, pharmacogenetics, adult-onset non-clinically actionable diseases, non-clinically actionable multifactorial diseases, and all genetic information) were significantly decreased after the workshop compared to beforehand (Appendix 3).

### Participants’ preferences regarding how to receive their genetic information by disease category before and after the genetics workshop

**Fig. 2:**
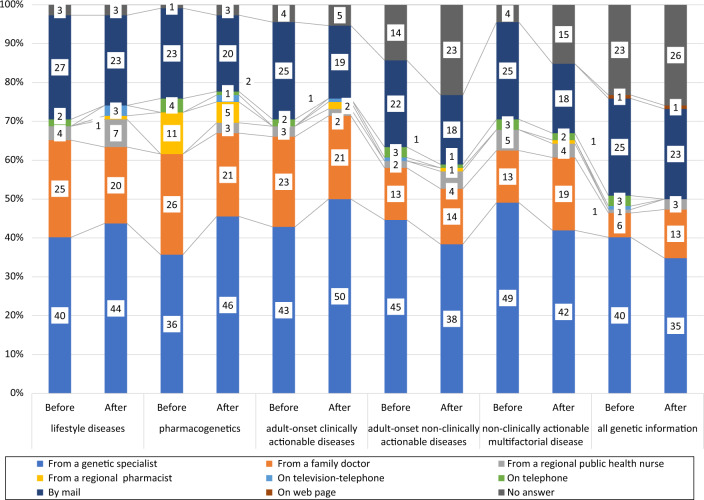
The participants’ preferred method of receiving their genetic information

Participants who preferred to receive their genetic results also provided information regarding how they preferred to receive their genetic information by disease category (Fig. 2). Most preferred to receive all genetic results from a genetic specialist, both before and after the genetics workshop. The numbers of participants who chose “From a genetic specialist” for lifestyle diseases, pharmacogenetics, and adult-onset clinically actionable diseases increased after the workshop compared to beforehand. For adult-onset non-clinically actionable diseases, non-clinically actionable diseases in which appearance of symptoms are not solely determined by genes, and all genetic information, the “No answer” rate increased after the workshop.

### Correlation between the participants’ genetic knowledge scores and preferences to receive their genetic information by disease category

There were no significant correlations between the total genetic knowledge scores and the participants’ preferences to receive their genetic information by disease category.

## Discussion

### Participant characteristics

In this study, nearly 90% of our participants were over 60 years old. This is consistent with our previous preference research based on residents of the Iwate and Miyagi prefectures, in which almost 76% of subjects were over 60 [6]. This may be because younger people are busy with work and child rearing, and could not find the time to participate in our genetics workshop. The use of a remote genetics workshop using a video or website could improve the response rate of younger people.

### Participants’ preferences to receive their genetic information by disease category before and after the genetics workshop

Over 95% of our participants answered “I want to know” or “If I must answer, then I want to know” for lifestyle diseases, pharmacogenetics, adult-onset clinically actionable diseases, and multifactorial diseases before the workshop, and lifestyle diseases, pharmacogenetics, and adult-onset clinically actionable diseases after the workshop. The highest rate of genetic result preference was 98%, both before and after the workshop. This preference rate was higher than the rates in the Tohoku Medical Megabank project’s participants (88%) and residents of the Tohoku area (82%) in a previous study [6]. More of our participants preferred to be informed of their genetic results regarding lifestyle diseases and pharmacogenetics than adult-onset non-clinically actionable diseases and all genetic information, both before and after the genetics workshop. The availability of disease treatment has been considered a deciding factor in preference for receiving genetic results [17]. However, in this study, treatment availability did not affect the preference to receive genetic results before the workshop. However, treatment availability may have been a deciding factor afterwards, except in the case of adult-onset clinically actionable diseases, as the preference to receive information in five of the six categories significantly decreased after the workshop. It might represent that participants perceived the information of adult-onset clinically actionable diseases as useful even after they gain additional genetic knowledge. A similar case for another disease is that for hypercholesterolemia, for which the Tohoku Medical Megabank Project has begun returning individual genetic information to the patients. Our participants also showed increased preference to receive genetic results regarding lifestyle diseases, pharmacogenetics, and adult-onset clinically actionable diseases from a genetic specialist after the workshop compared to beforehand. The “No answer” rate regarding how to receive genetic information was increased for adult-onset non-clinically actionable diseases, non-clinically actionable multifactorial diseases, and all genetic information. This tendency may represent hesitation in our participants regarding the receipt of this information after gaining accurate knowledge of human genetics at the genetics workshop.

### Comparison of participants’ genetic knowledge before and after the genetics workshop

The total score of our participants’ genetic knowledge was significantly increased despite the fact that nearly 90% of the participants were over 60 years, and it is considered that elder people find it relatively difficult to learn new knowledge. There was a positive correlation between the total genetic knowledge score and the participants’ educational background level before the genetics workshop. However, after the genetics workshop, there were no significant correlations between these variables. This suggests that, regardless of their educational background, people could increase their genetic knowledge by attending the workshop. This is important, as genetic education in Japan has been non-uniformly developed, and individual differences and those between generations in genetic literacy are relatively large. There exist changes in educational curriculum among different age groups. For example, Mendelian genetics were taught in junior high school until the 1970s; however, this content disappeared from the curriculum. Mendelian genetics taught in junior high school reappeared in the textbook in 2012. The concept of the gene was also taught in the 1970s, disappeared from the curriculum, and reappeared in the late 2000s [18]. Basic knowledge of molecular genetics, inheritance, and species variation has generally increased but information on human genetics, especially disease-related concepts, is minimal in the latest government curriculum guidelines [19–21]. Harper reported that cultural isolation and extreme sensitivity over family matters, including genetic disorders, may have been delaying factors for the dissemination of medical genetics knowledge in Japan [22]. It is also known that educational backgrounds were different among the age groups. High school qualification constituted the highest percentage of educational background across all age groups in Japanese population census and among our participants. However, people in younger age groups had higher educational qualifications (Appendix 4-1). The second highest percentage of educational background was university undergraduate and/or graduate degrees (18.5%) across all ages of Japanese population census (Appendix 4-2), primary and/or junior high school qualification (28.8%) for age groups within the range of 60 to 79 years in the census (Appendix 4-3), and vocational and/or junior college qualification (22.4%) among our participants (Table 2). Accurate knowledge of human genetics is the foundation of personalized genetic medicine. Our workshop could be an opportunity to homogenize study participants’ genetic knowledge and help participants understand their genetic results.

### Study limitations

There are several limitations to our study. First, our findings may have been influenced by response bias, as attending the genetics workshop demanded participant time and effort, which might have been considered inconvenient by younger and early-middle-aged people and thus created a potential disincentive for them to participate in our study. Our participants’ distribution was therefore biased toward older people for whom it was relatively easy to take time to attend the workshop, and this led to increased attendance indicating that our participants may have been motivated and interested to understand their genetic information. For future research, offering a web-based genetic workshop and questionnaire could improve the response rate of those people. Second, we conducted the second questionnaire soon after the genetics workshop, and the long-term effects of the genetics workshop were not assessed. Third, all participants lived in the Tohoku region, and we did not include other areas in Japan for this study. As such, our results may not be generalizable to other regions and ages in Japan.

## Electronic supplementary material

Appendix1-1slide1

Appendix1-1slide2

Appendix1-1slide3

Appendix1-1slide4

Appendix1-1slide5

Appendix1-1slide6

Appendix1-1slide7

Appendix1-1slide8

Appendix1-1slide9

Appendix1-1slide10

Appendix1-1slide11

Appendix1-1slide12

Appendix1-1slide13

Appendix1-1slide14

Appendix1-1slide15

Appendix1-1slide16

Appendix1-1slide17

Appendix1-1slide18

Appendix1-1slide19

Appendix1-1slide20

Appendix1-1slide21

Appendix1-1slide22

Appendix1-1slide23

Appendix1-1slide24

Appendix1-1slide25

Appendix1-1slide26

Appendix1-1slide27

Appendix1-1slide28

Appendix1-1slide29

Appendix1-1slide30

Appendix1-1slide31

Appendix1-1slide32

Appendix1-1slide33

Appendix1-1slide34

Appendix1-1slide35

Appendix 1-2

Appendix 2

Appendix 3

Appendix 4-1

Appendix 4-2

Appendix 4-3
