# Supplementary figures and images for: A comparison of genome cohort participants’ genetic knowledge and preferences to receive genetic results before and after a genetics workshop
Source: J Hum Genet. 2018 Sep 5;63(11):1139–47. doi: 10.1038/s10038-018-0494-z (PMC8075932; doi:10.1038/s10038-018-0494-z)

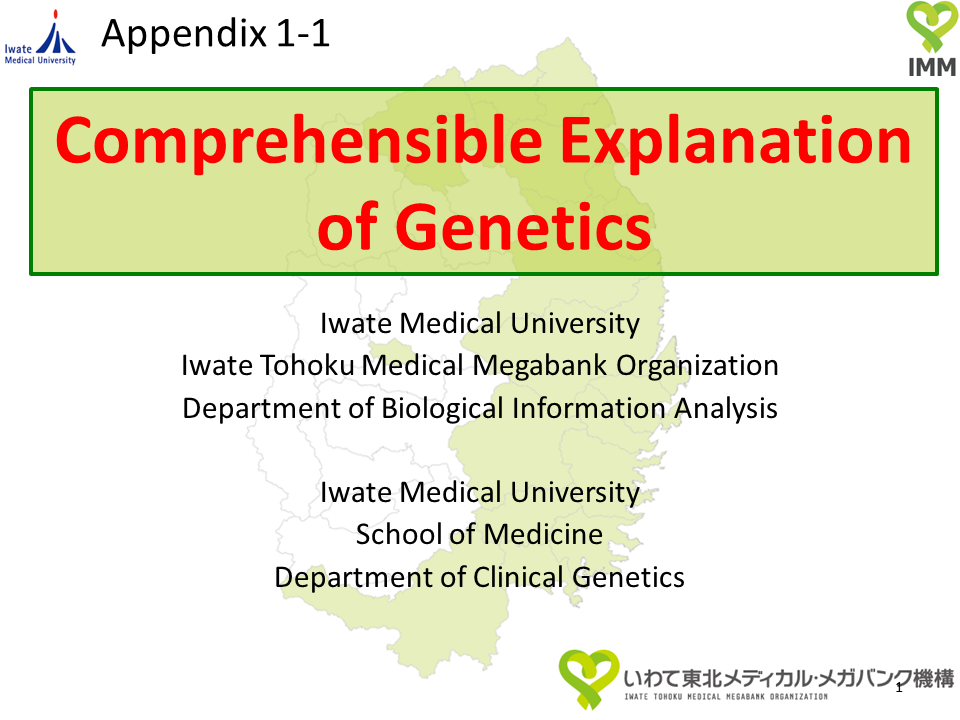

Supplement: Supplementary file 1 — Appendix1-1slide1 [file 10038_2018_494_MOESM1_ESM.tif]

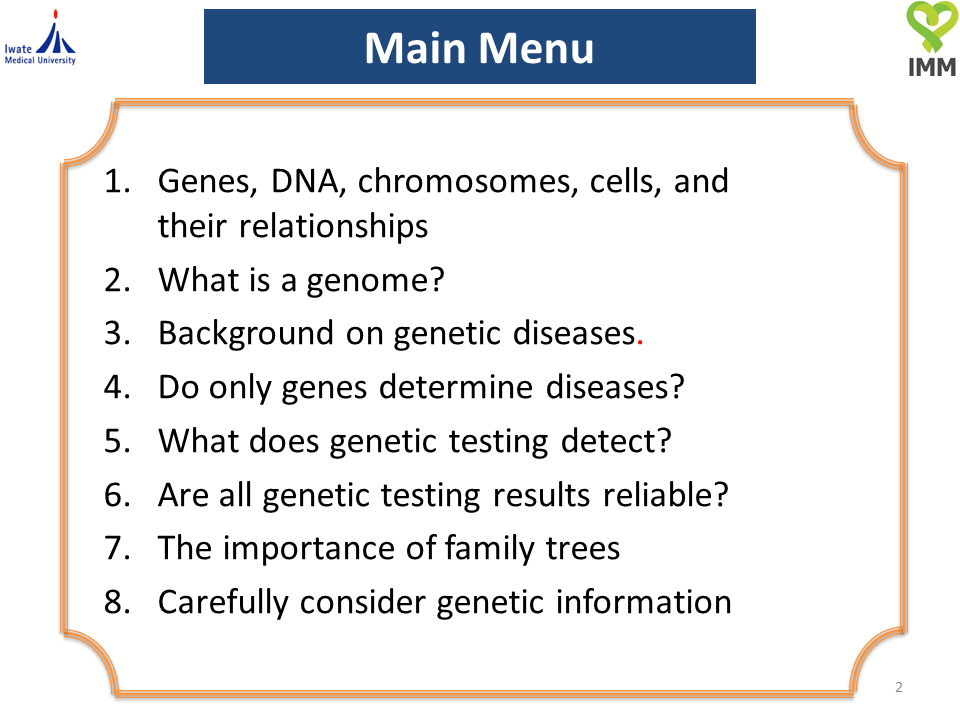

Supplement: Supplementary file 2 — Appendix1-1slide2 [file 10038_2018_494_MOESM2_ESM.tif]

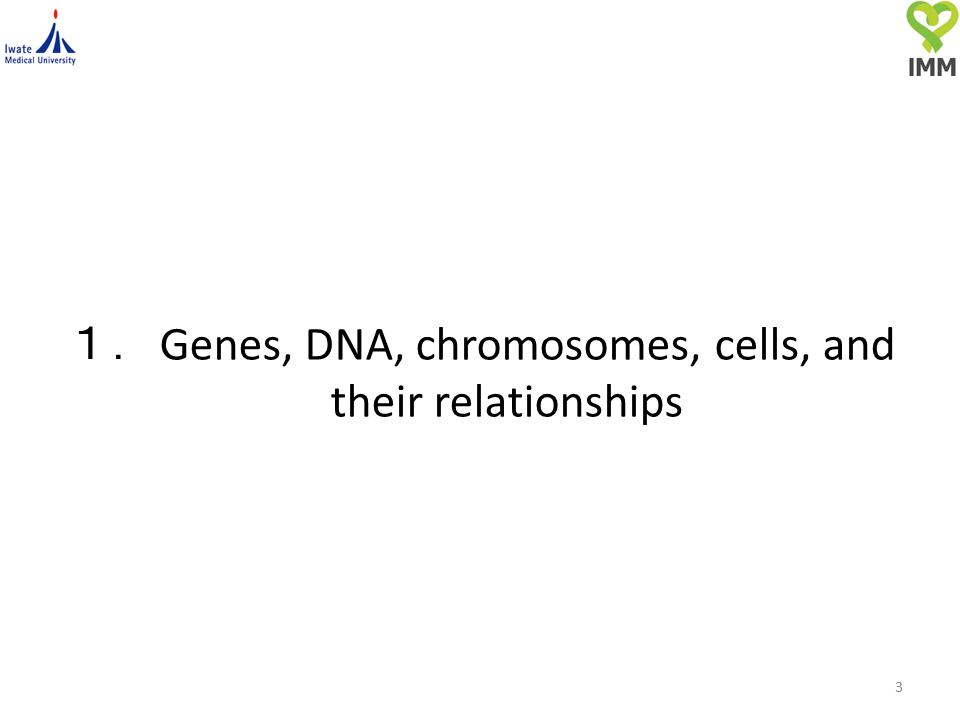

Supplement: Supplementary file 3 — Appendix1-1slide3 [file 10038_2018_494_MOESM3_ESM.tif]

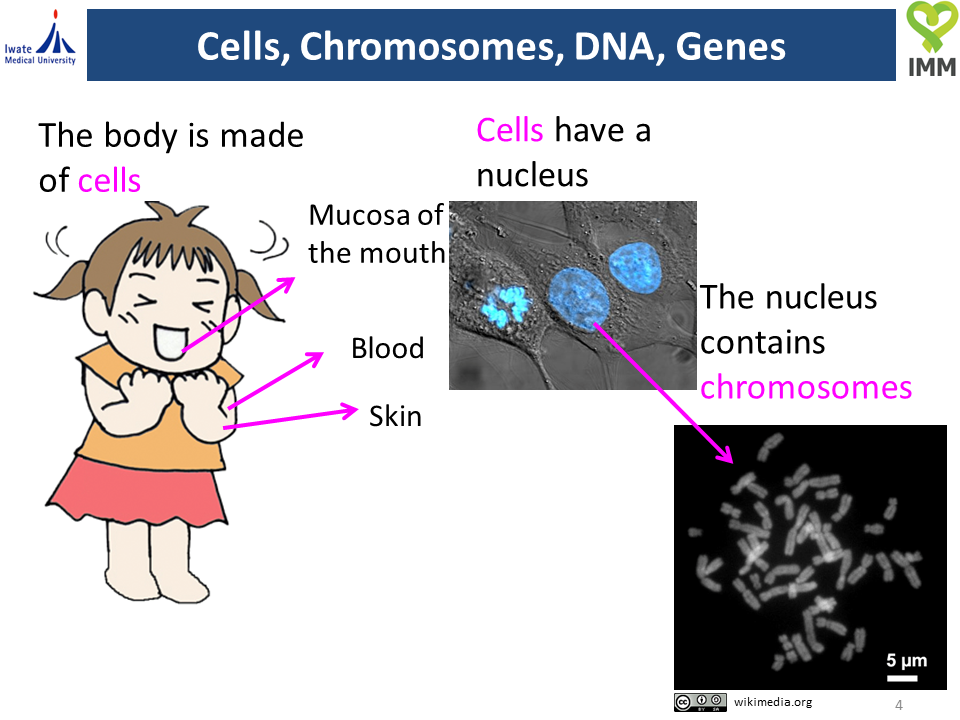

Supplement: Supplementary file 4 — Appendix1-1slide4 [file 10038_2018_494_MOESM4_ESM.tif]

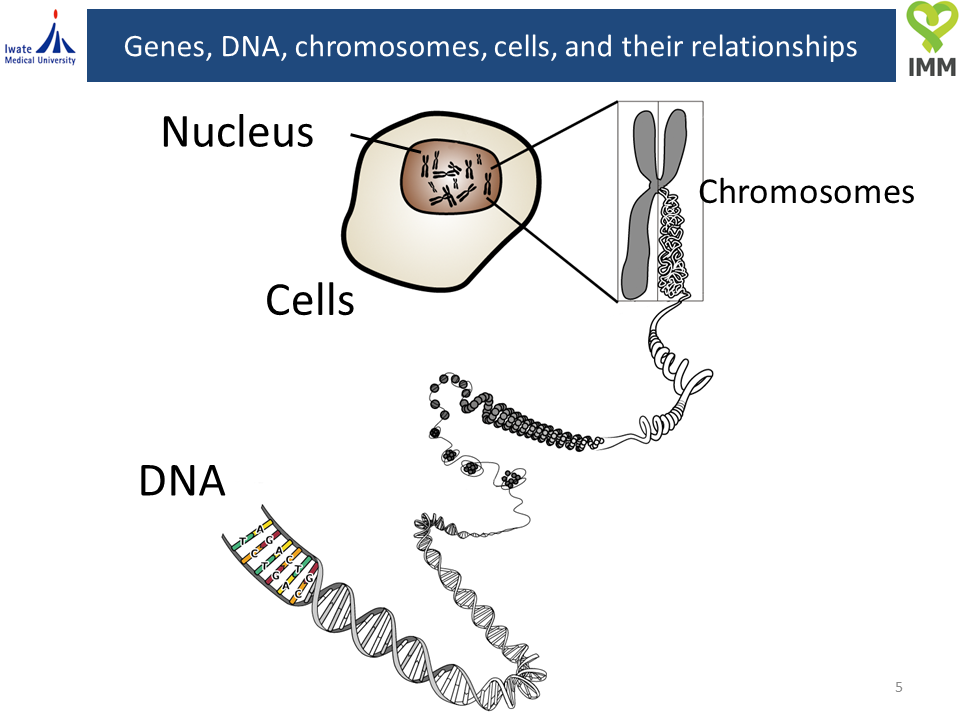

Supplement: Supplementary file 5 — Appendix1-1slide5 [file 10038_2018_494_MOESM5_ESM.tif]

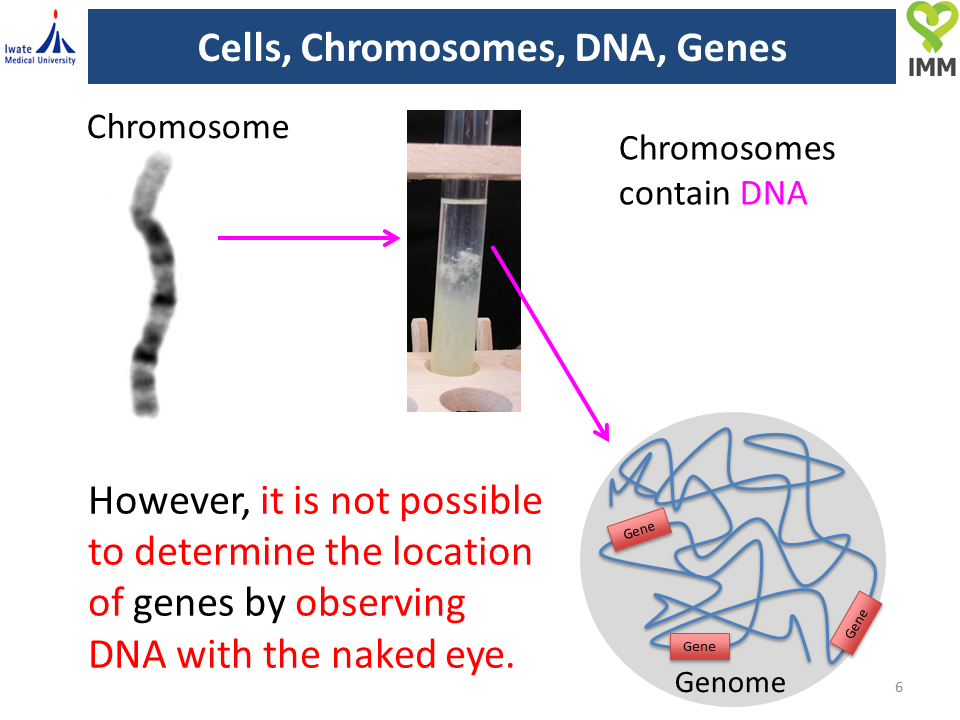

Supplement: Supplementary file 6 — Appendix1-1slide6 [file 10038_2018_494_MOESM6_ESM.tif]

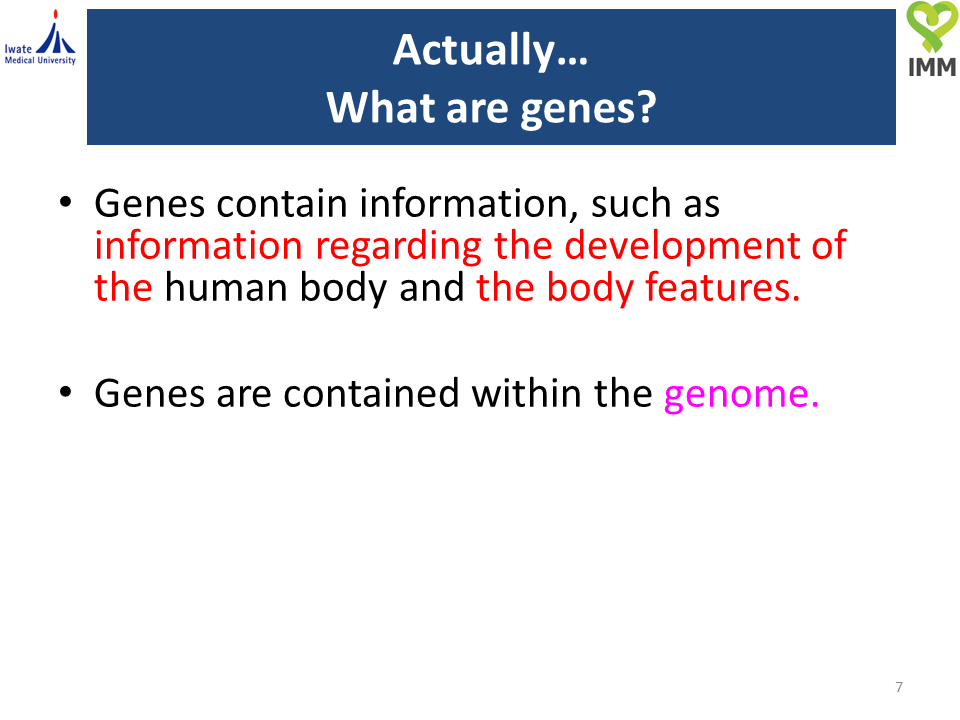

Supplement: Supplementary file 7 — Appendix1-1slide7 [file 10038_2018_494_MOESM7_ESM.tif]

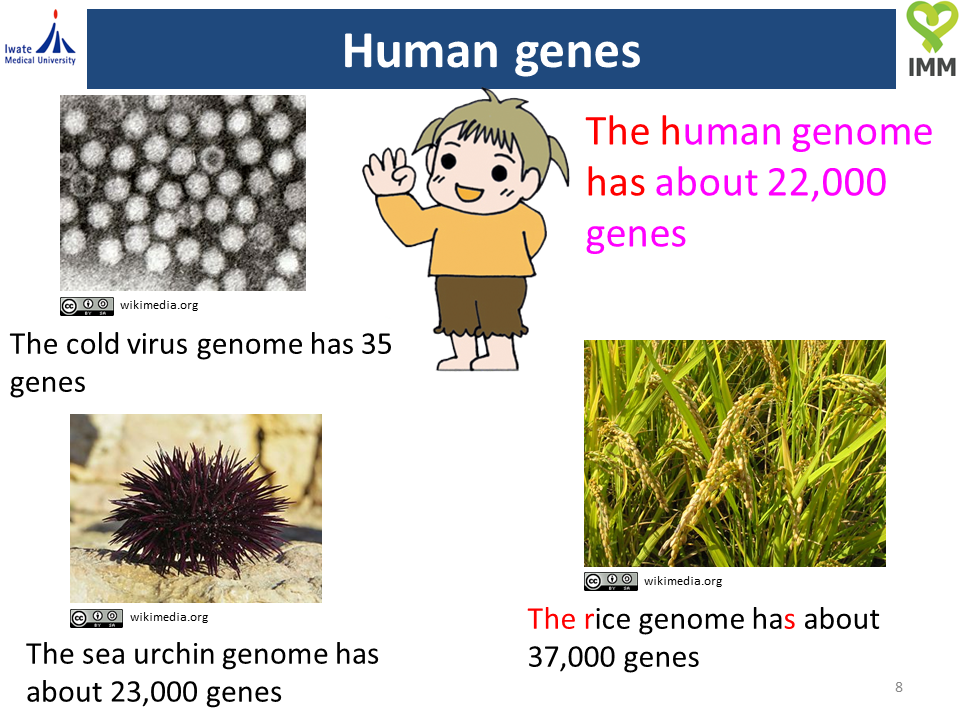

Supplement: Supplementary file 8 — Appendix1-1slide8 [file 10038_2018_494_MOESM8_ESM.tif]

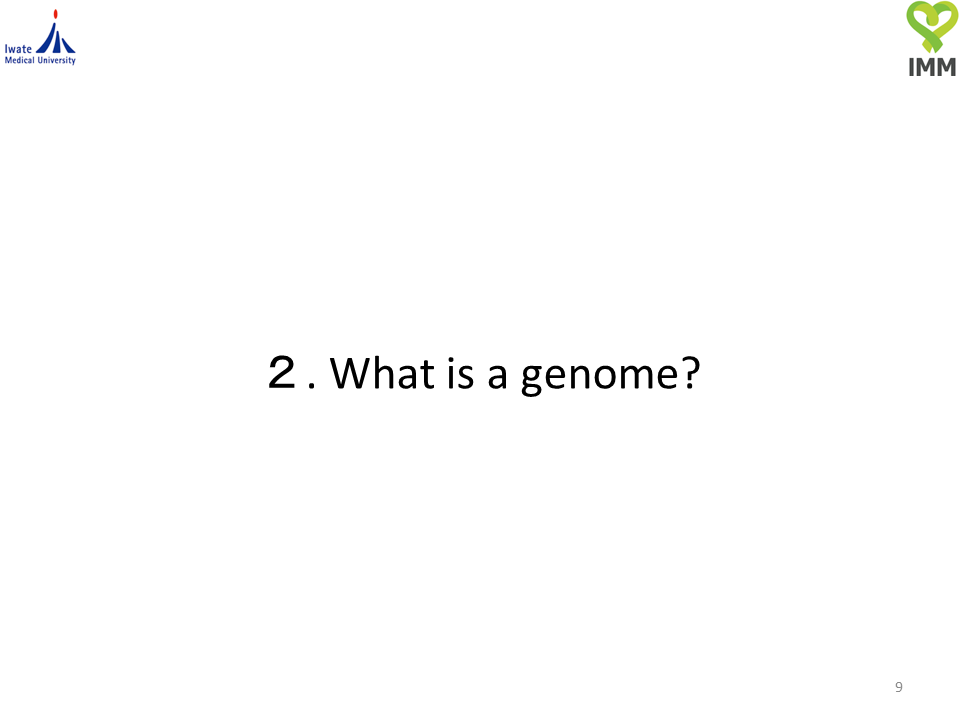

Supplement: Supplementary file 9 — Appendix1-1slide9 [file 10038_2018_494_MOESM9_ESM.tif]

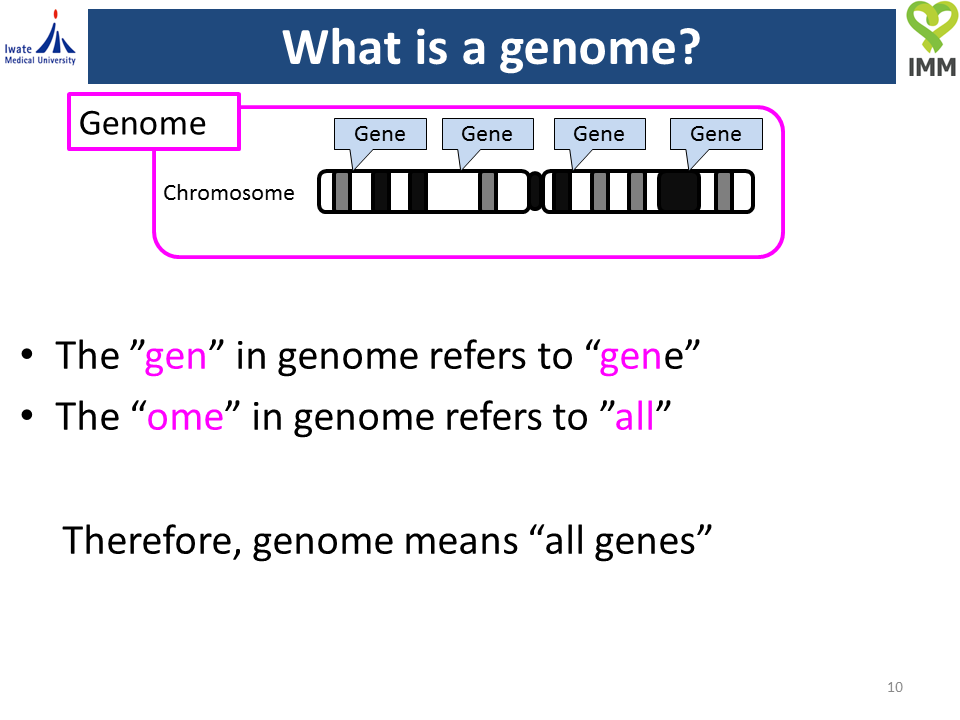

Supplement: Supplementary file 10 — Appendix1-1slide10 [file 10038_2018_494_MOESM10_ESM.tif]

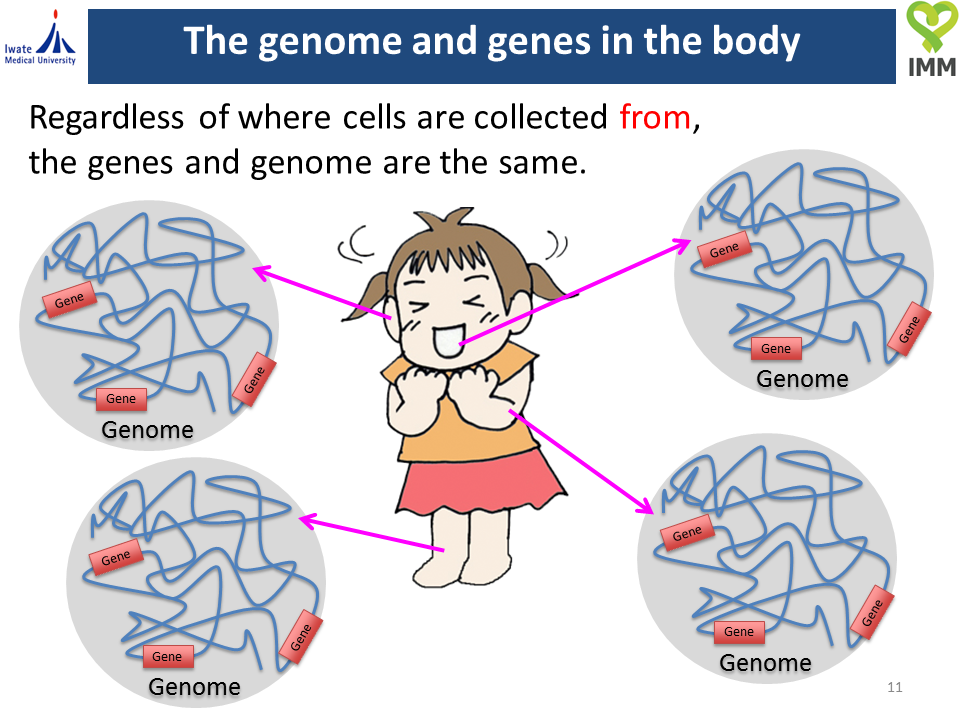

Supplement: Supplementary file 11 — Appendix1-1slide11 [file 10038_2018_494_MOESM11_ESM.tif]

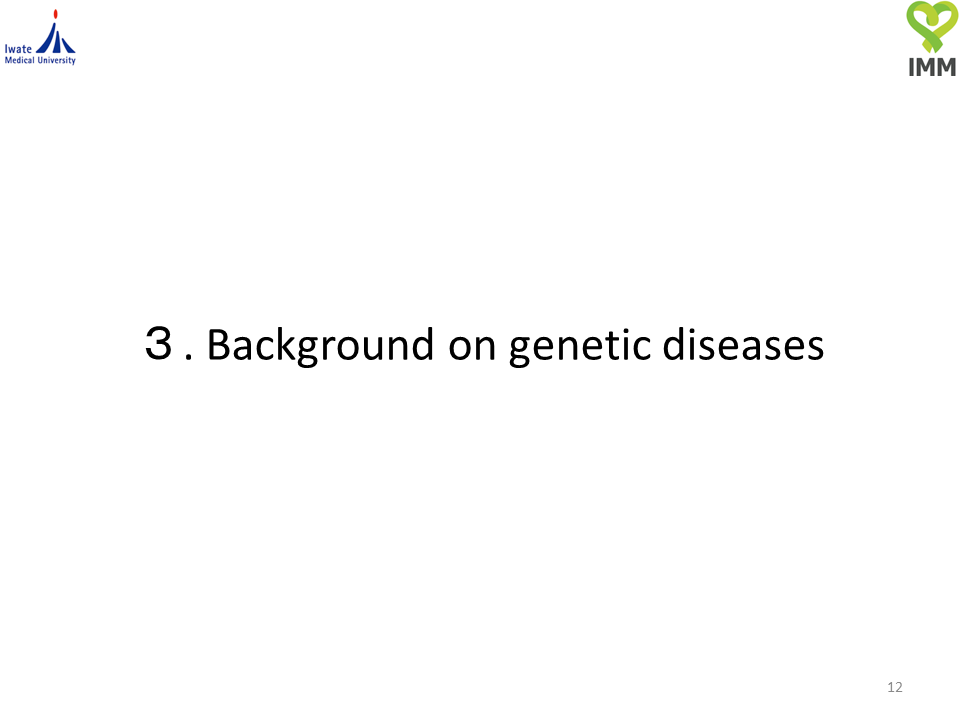

Supplement: Supplementary file 12 — Appendix1-1slide12 [file 10038_2018_494_MOESM12_ESM.tif]

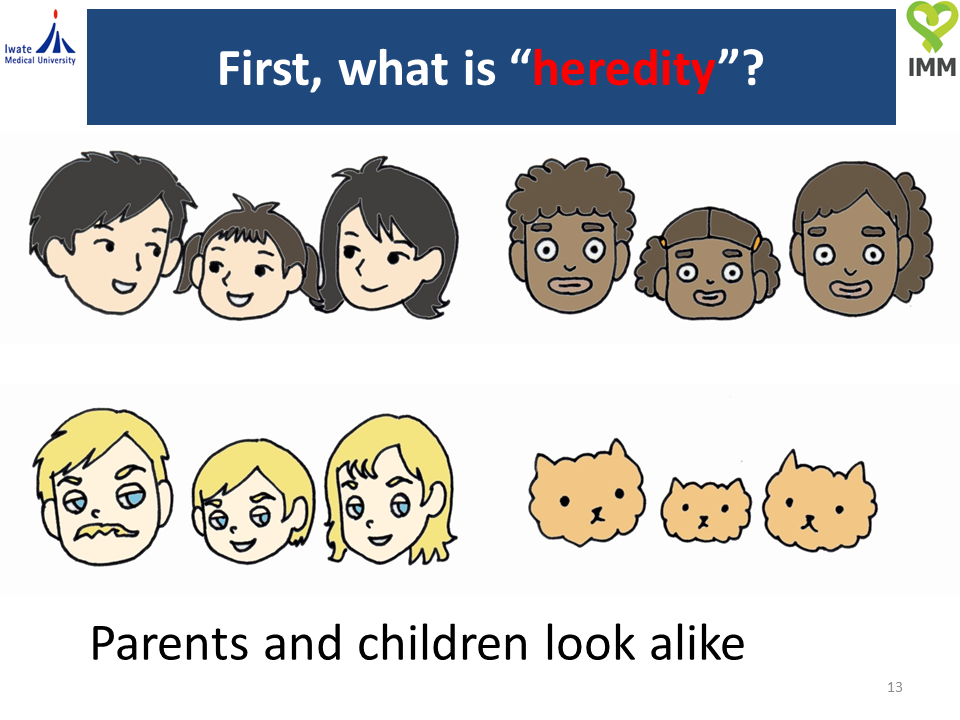

Supplement: Supplementary file 13 — Appendix1-1slide13 [file 10038_2018_494_MOESM13_ESM.tif]

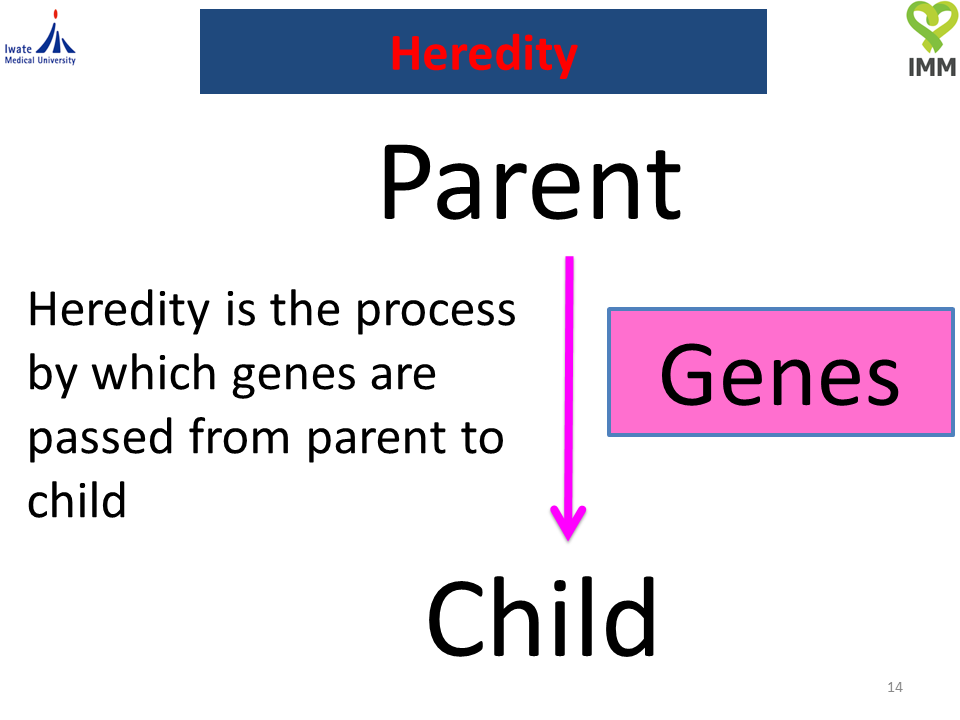

Supplement: Supplementary file 14 — Appendix1-1slide14 [file 10038_2018_494_MOESM14_ESM.tif]

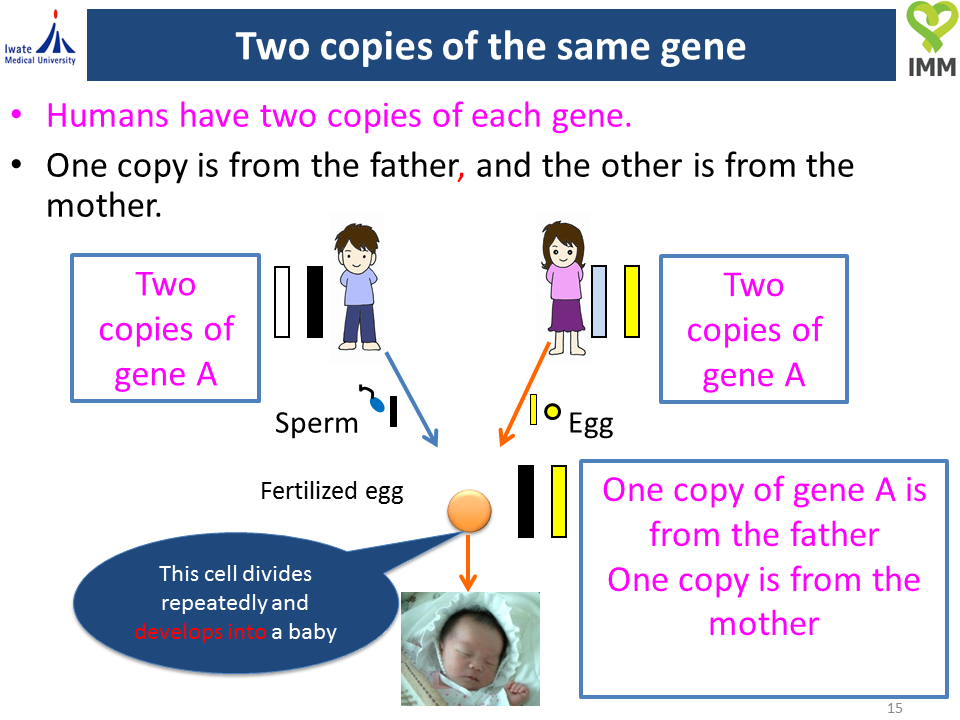

Supplement: Supplementary file 15 — Appendix1-1slide15 [file 10038_2018_494_MOESM15_ESM.tif]

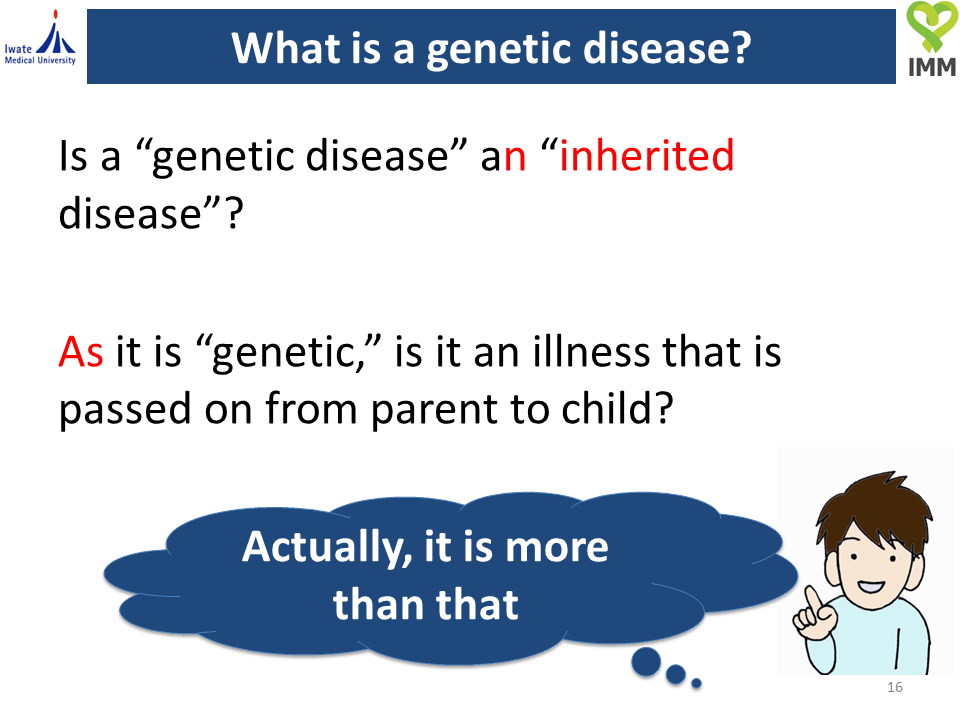

Supplement: Supplementary file 16 — Appendix1-1slide16 [file 10038_2018_494_MOESM16_ESM.tif]

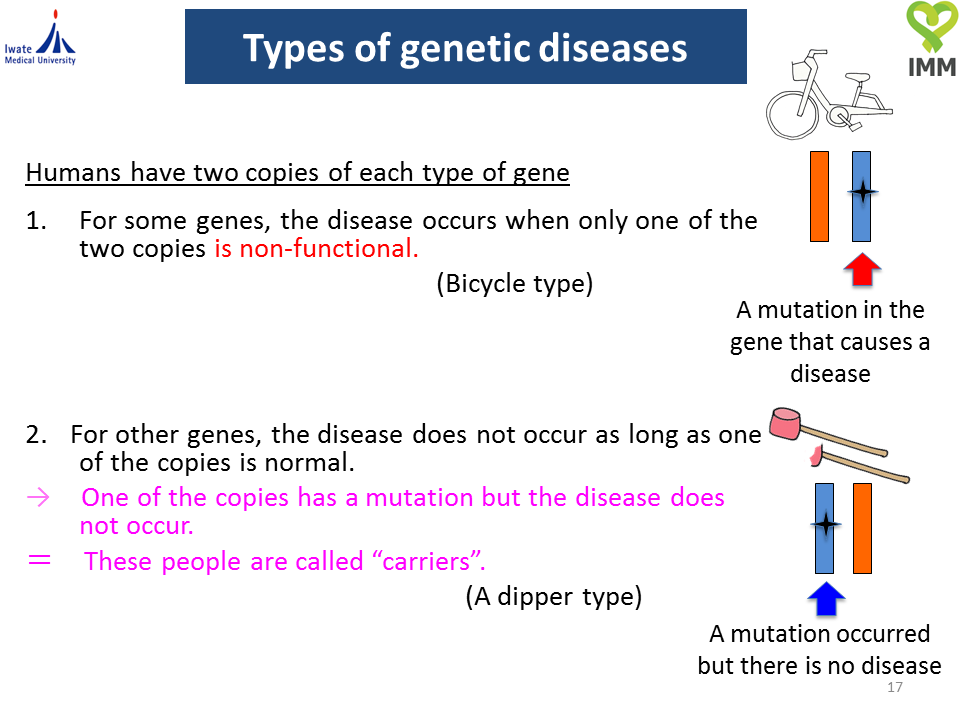

Supplement: Supplementary file 17 — Appendix1-1slide17 [file 10038_2018_494_MOESM17_ESM.tif]

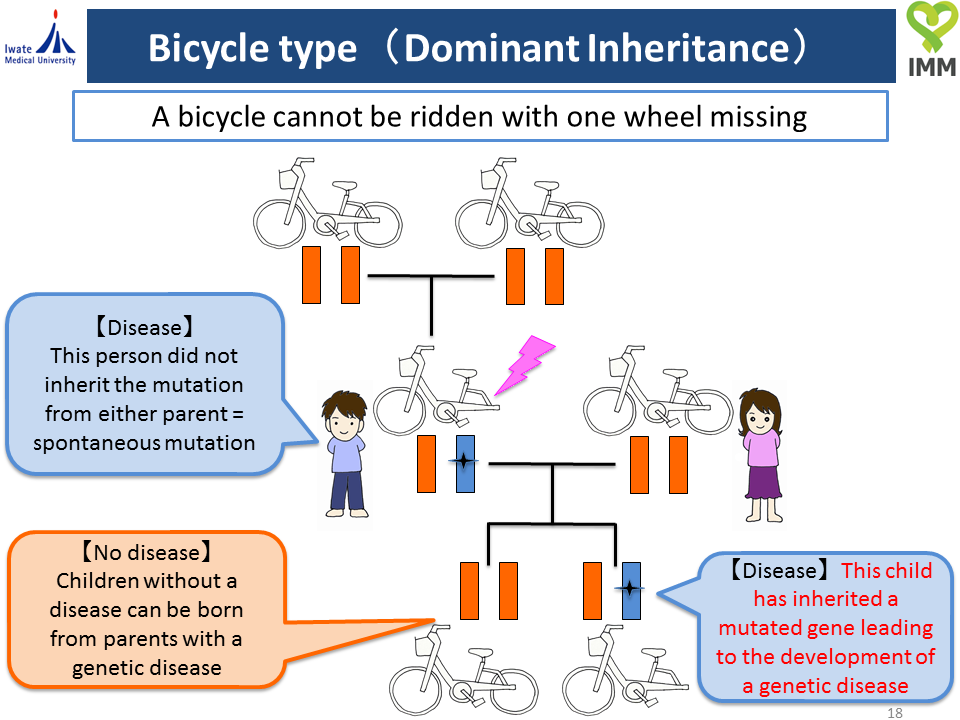

Supplement: Supplementary file 18 — Appendix1-1slide18 [file 10038_2018_494_MOESM18_ESM.tif]

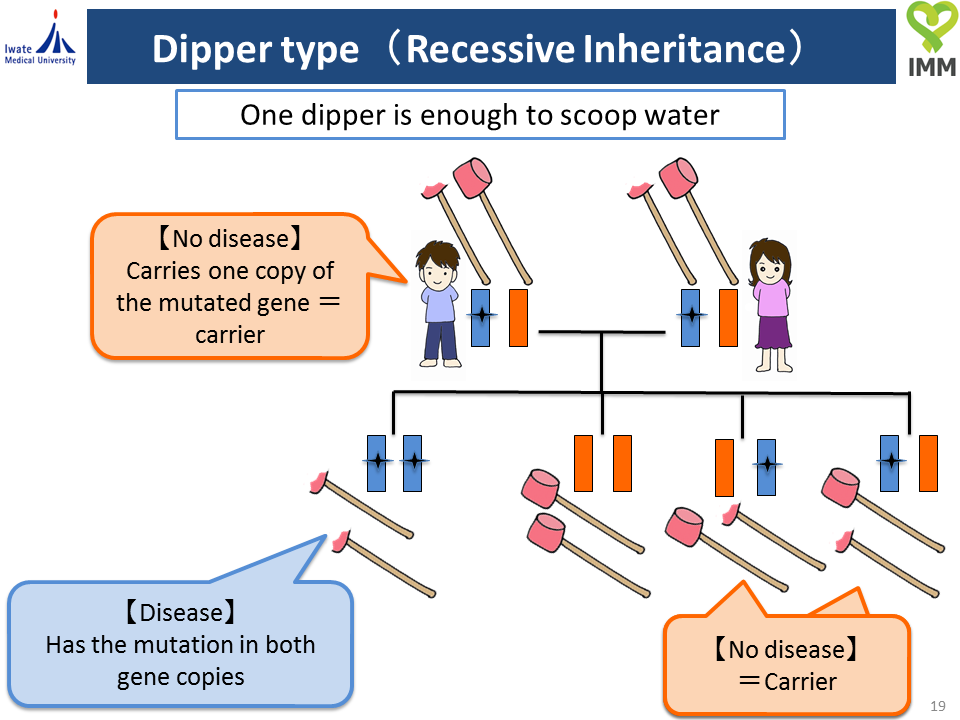

Supplement: Supplementary file 19 — Appendix1-1slide19 [file 10038_2018_494_MOESM19_ESM.tif]

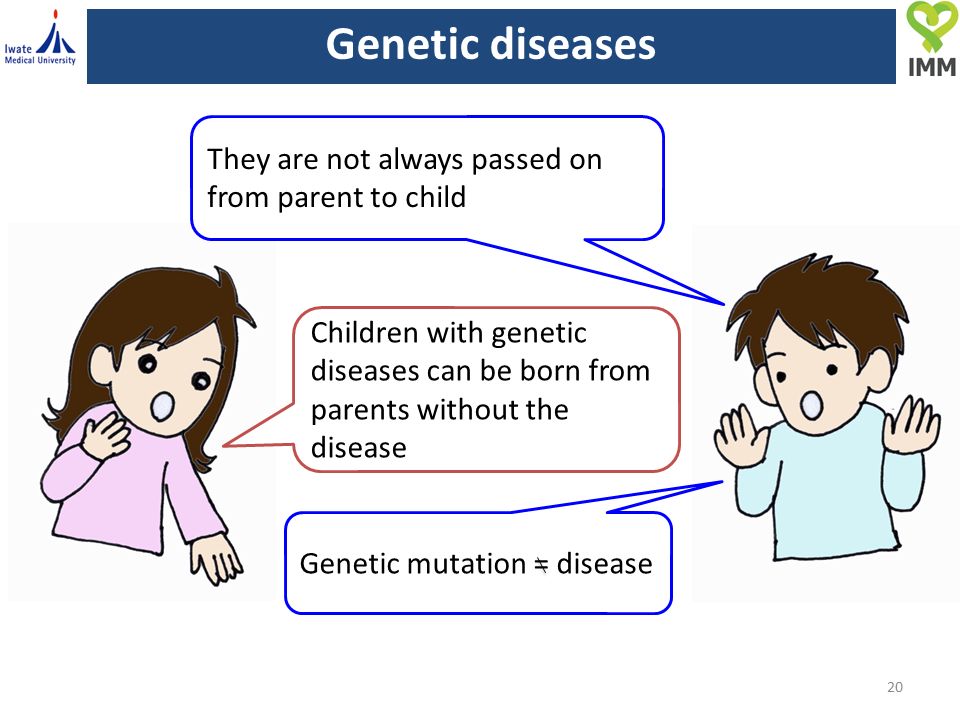

Supplement: Supplementary file 20 — Appendix1-1slide20 [file 10038_2018_494_MOESM20_ESM.tif]

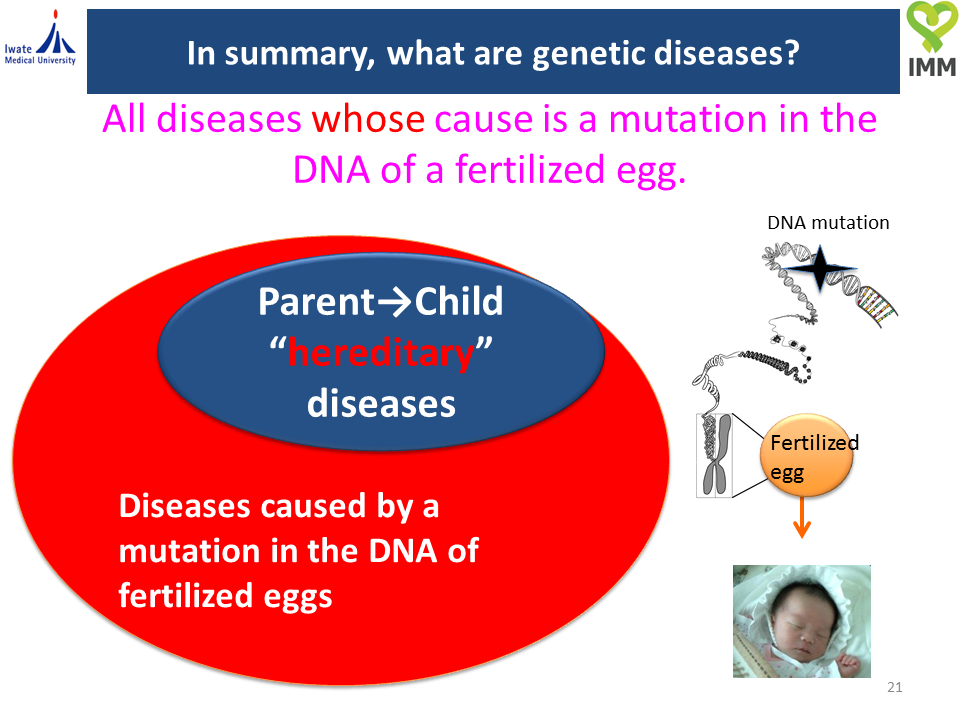

Supplement: Supplementary file 21 — Appendix1-1slide21 [file 10038_2018_494_MOESM21_ESM.tif]

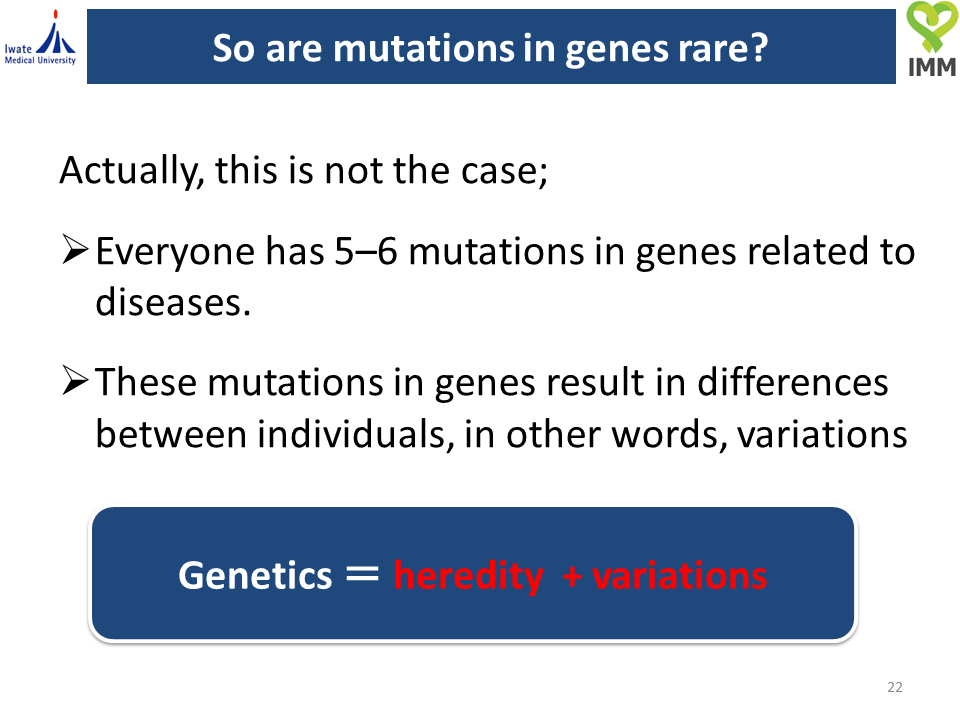

Supplement: Supplementary file 22 — Appendix1-1slide22 [file 10038_2018_494_MOESM22_ESM.tif]

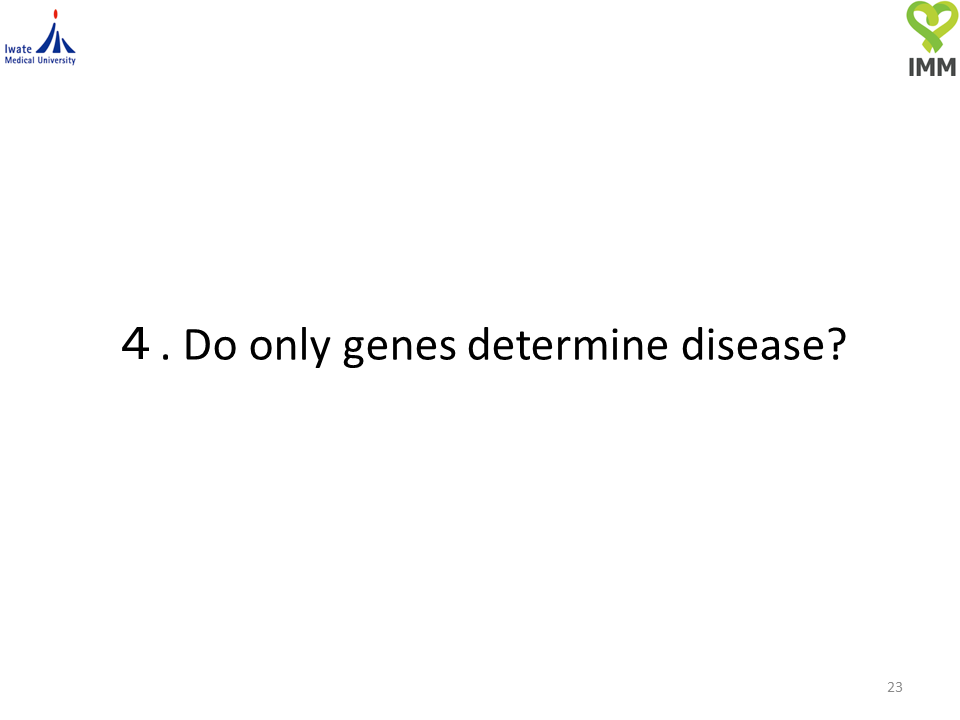

Supplement: Supplementary file 23 — Appendix1-1slide23 [file 10038_2018_494_MOESM23_ESM.tif]

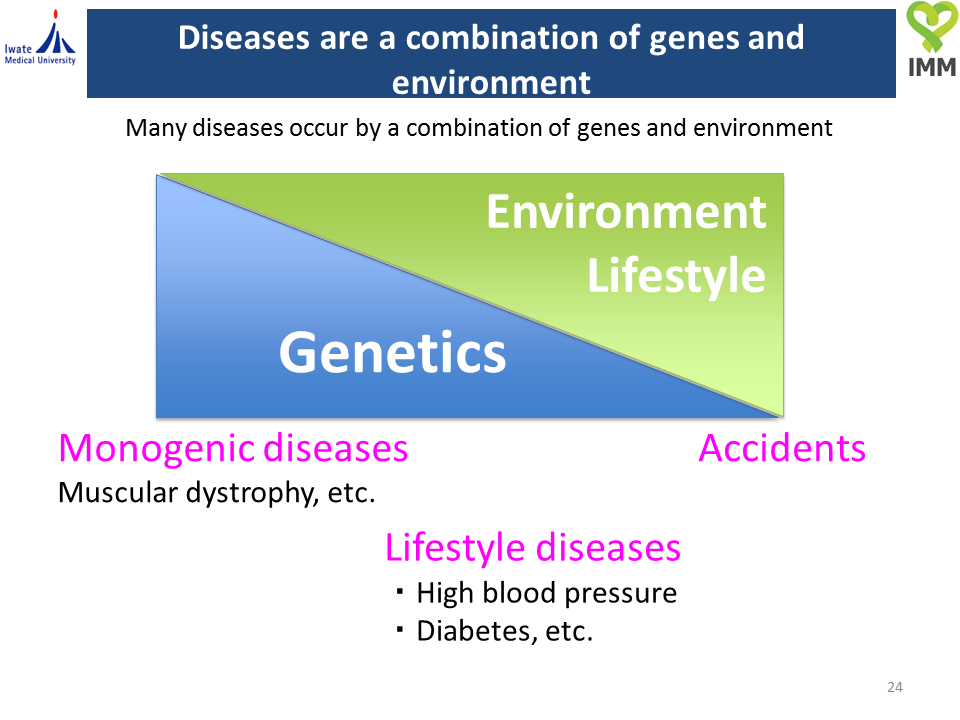

Supplement: Supplementary file 24 — Appendix1-1slide24 [file 10038_2018_494_MOESM24_ESM.tif]

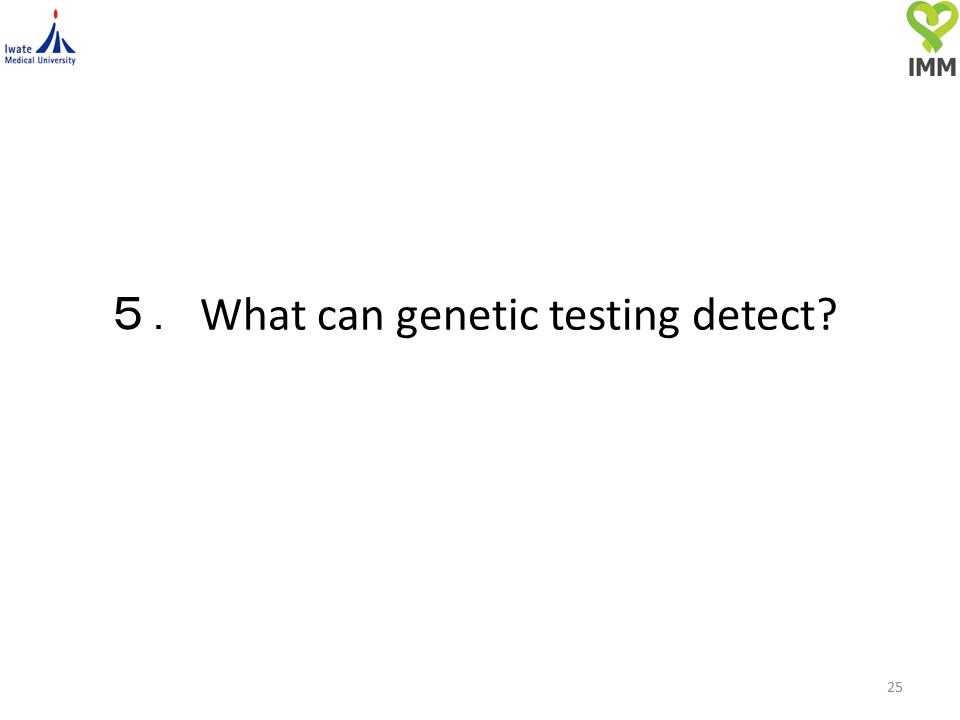

Supplement: Supplementary file 25 — Appendix1-1slide25 [file 10038_2018_494_MOESM25_ESM.tif]

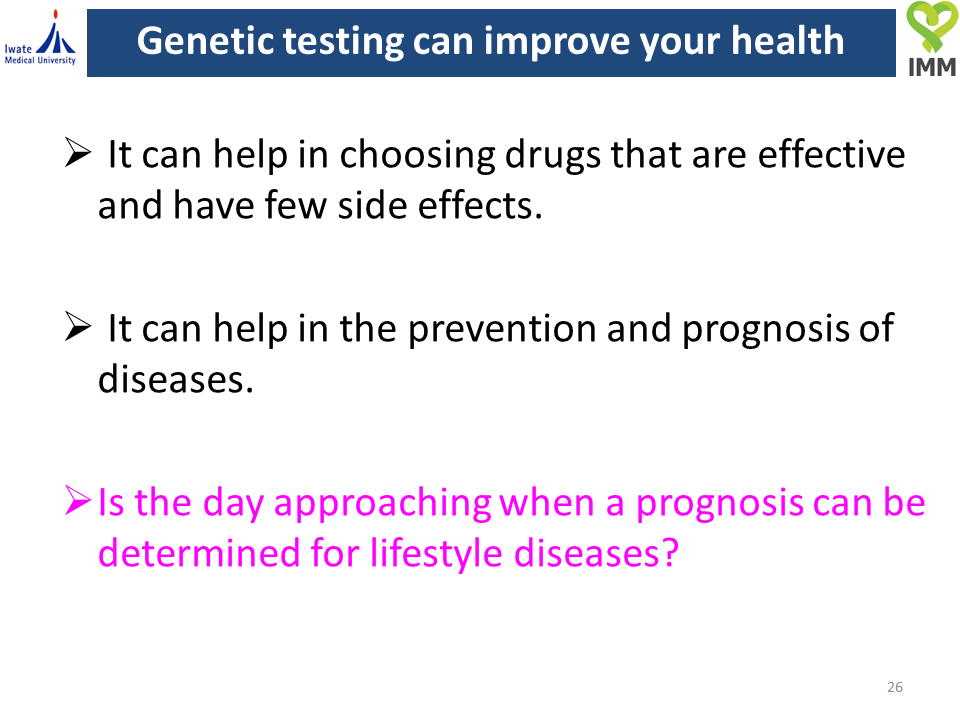

Supplement: Supplementary file 26 — Appendix1-1slide26 [file 10038_2018_494_MOESM26_ESM.tif]

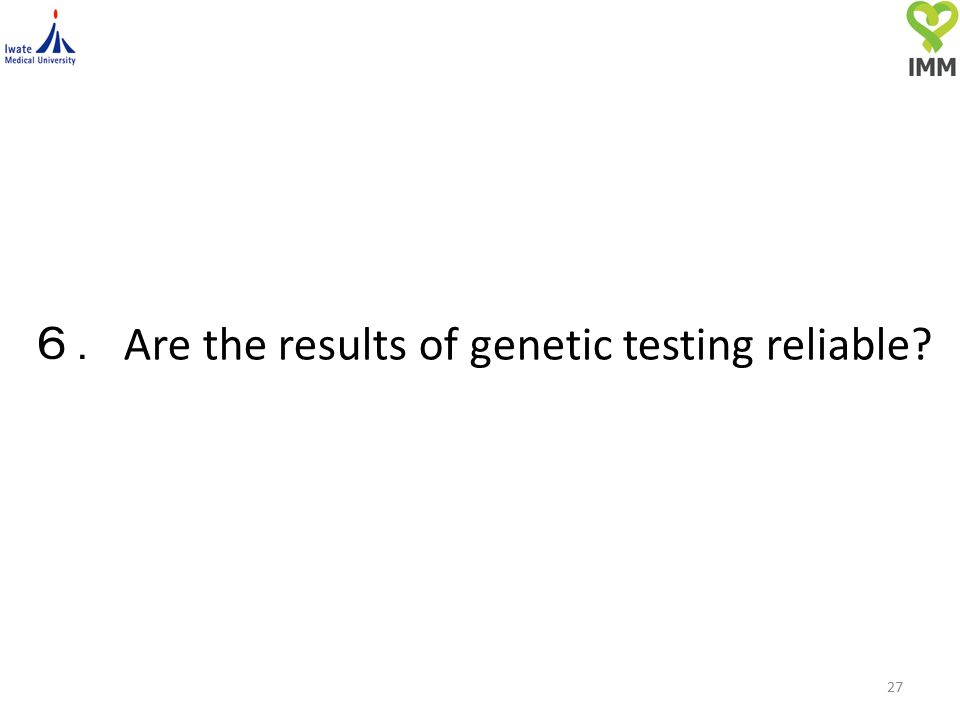

Supplement: Supplementary file 27 — Appendix1-1slide27 [file 10038_2018_494_MOESM27_ESM.tif]

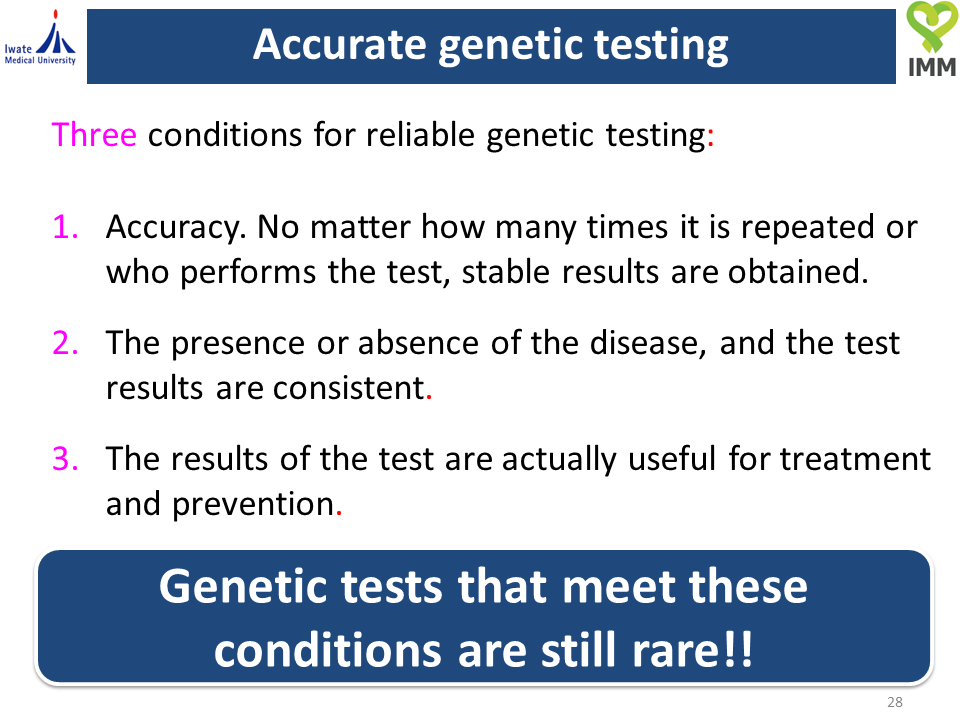

Supplement: Supplementary file 28 — Appendix1-1slide28 [file 10038_2018_494_MOESM28_ESM.tif]

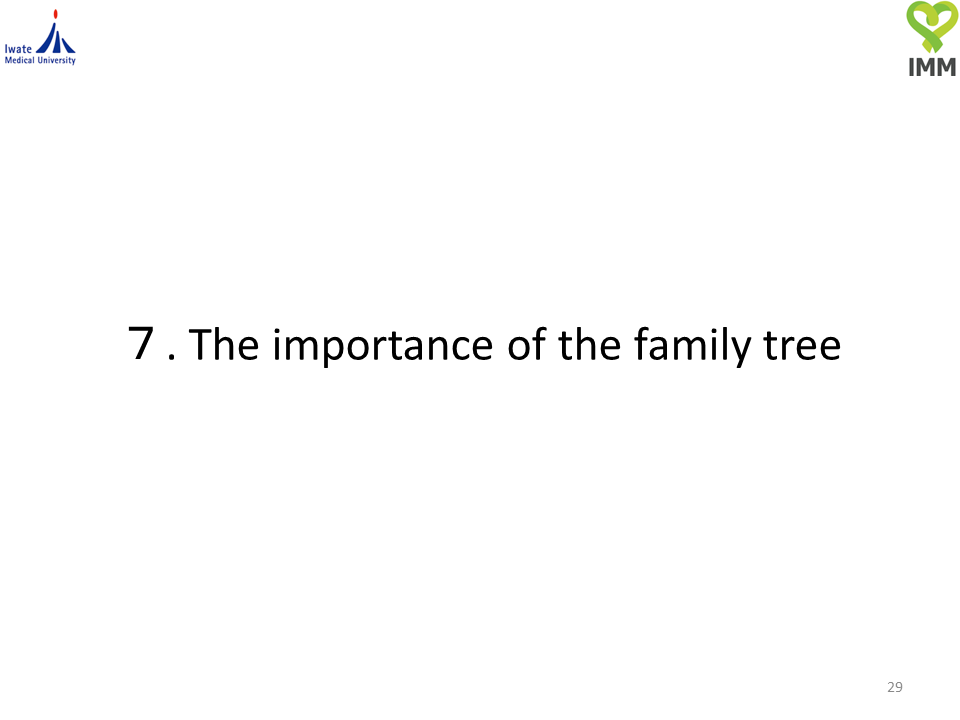

Supplement: Supplementary file 29 — Appendix1-1slide29 [file 10038_2018_494_MOESM29_ESM.tif]

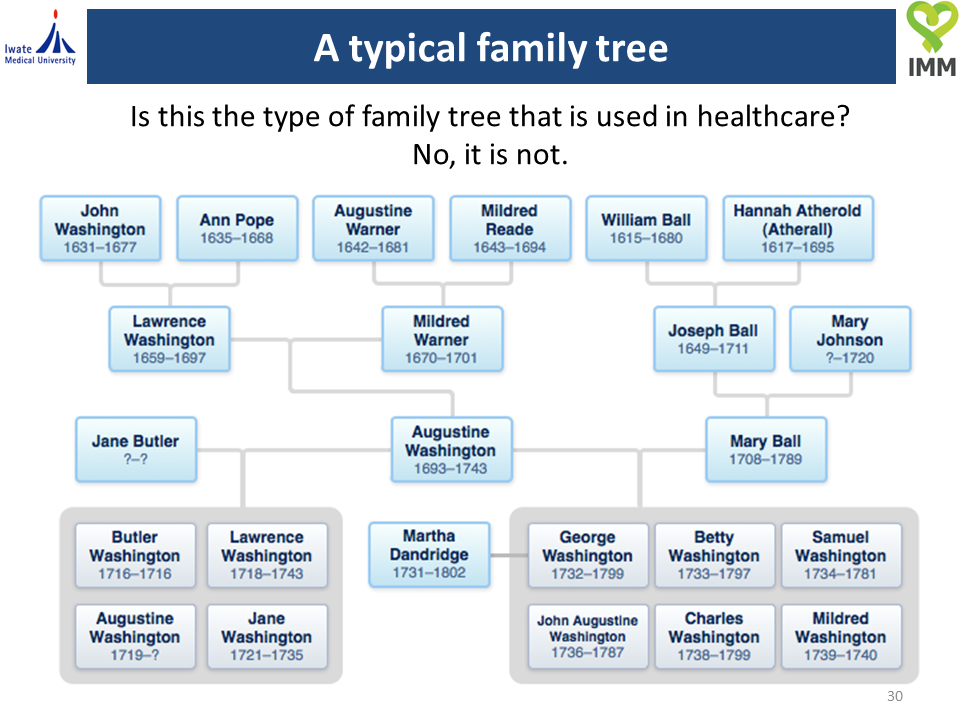

Supplement: Supplementary file 30 — Appendix1-1slide30 [file 10038_2018_494_MOESM30_ESM.tif]

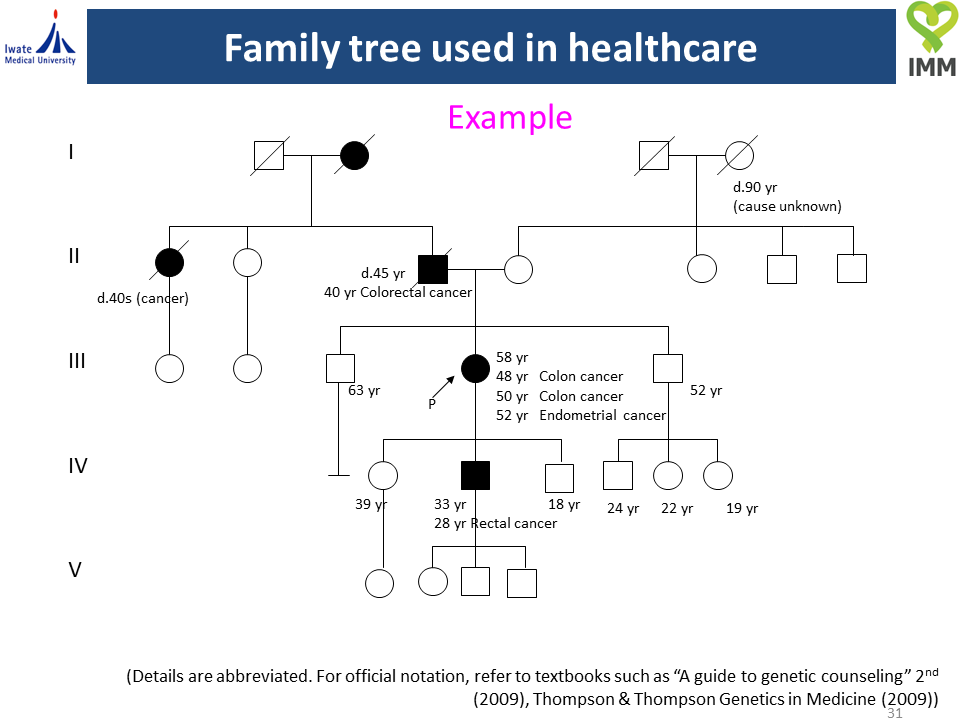

Supplement: Supplementary file 31 — Appendix1-1slide31 [file 10038_2018_494_MOESM31_ESM.tif]

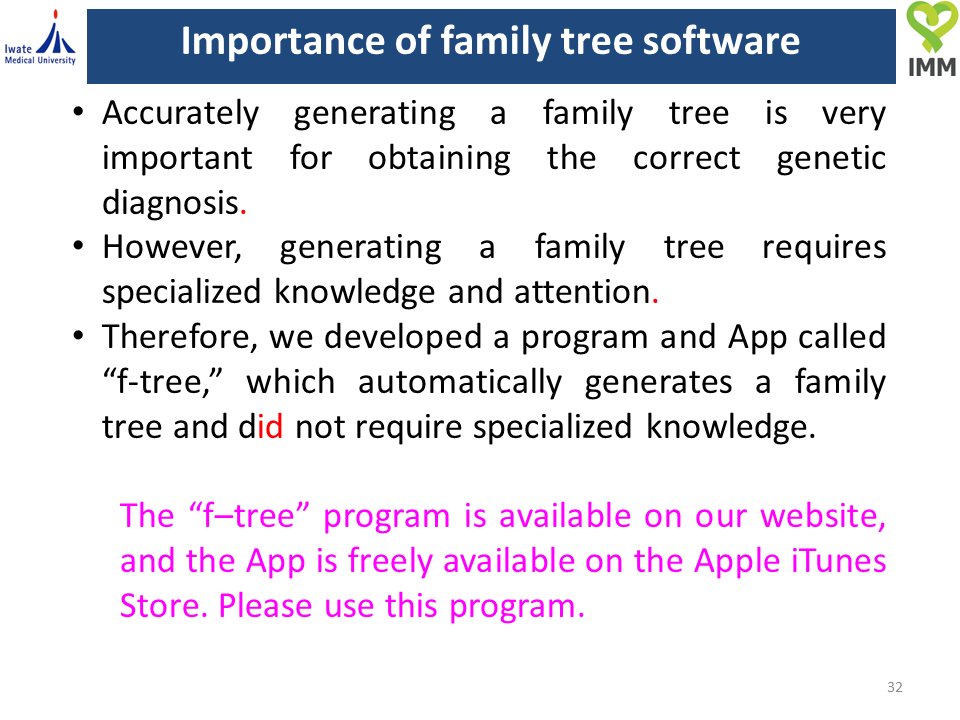

Supplement: Supplementary file 32 — Appendix1-1slide32 [file 10038_2018_494_MOESM32_ESM.tif]

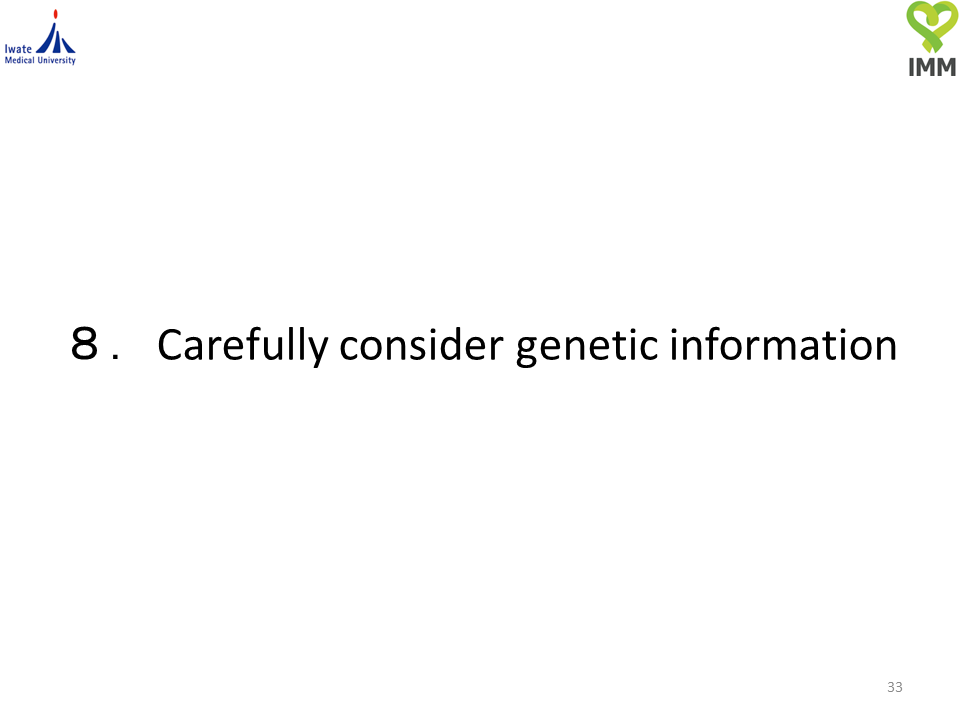

Supplement: Supplementary file 33 — Appendix1-1slide33 [file 10038_2018_494_MOESM33_ESM.tif]

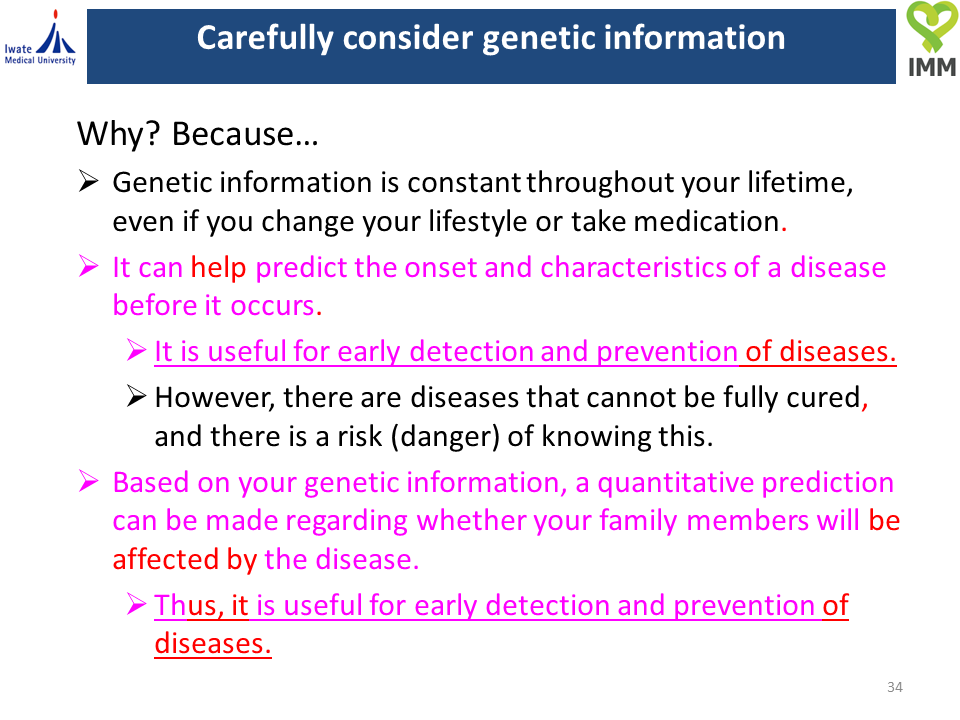

Supplement: Supplementary file 34 — Appendix1-1slide34 [file 10038_2018_494_MOESM34_ESM.tif]

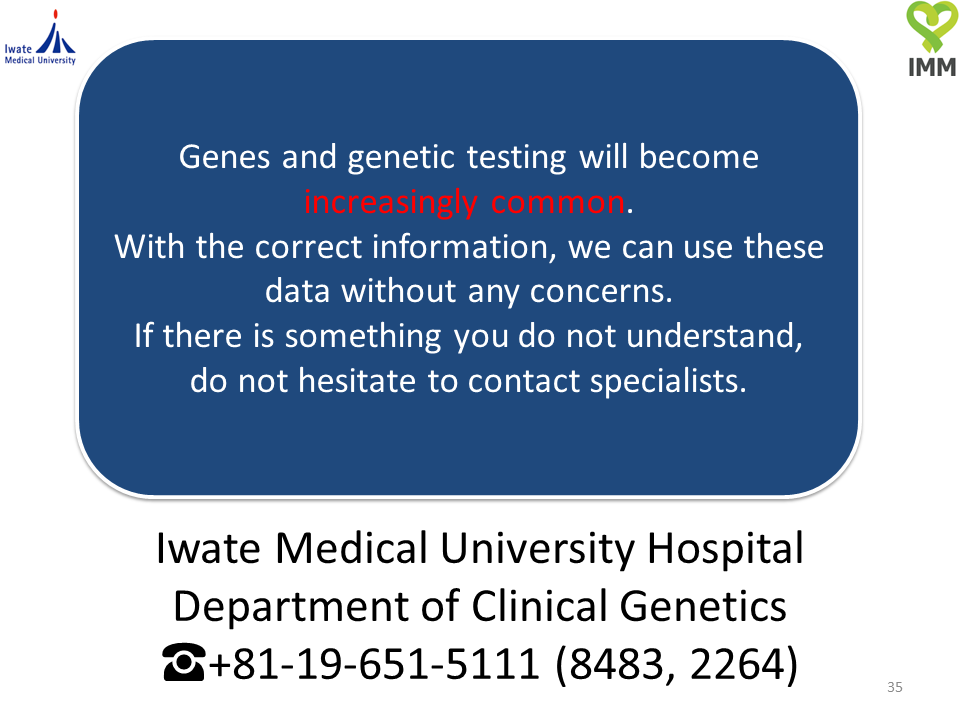

Supplement: Supplementary file 35 — Appendix1-1slide35 [file 10038_2018_494_MOESM35_ESM.tif]

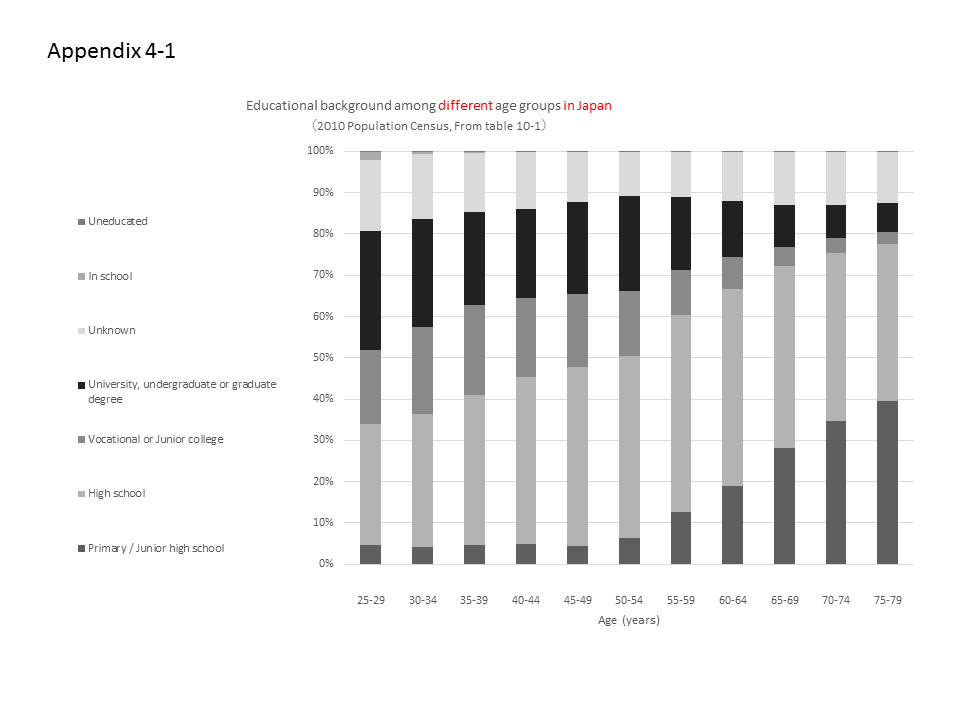

Supplement: Supplementary file 39 — Appendix 4-1 [file 10038_2018_494_MOESM39_ESM.tif]

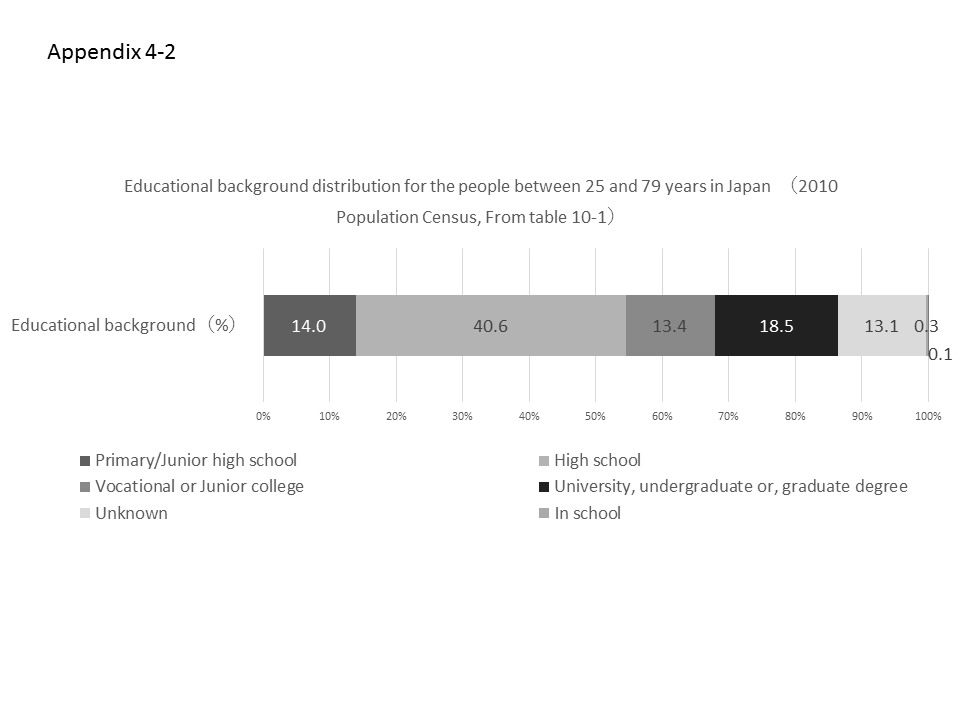

Supplement: Supplementary file 40 — Appendix 4-2 [file 10038_2018_494_MOESM40_ESM.tif]

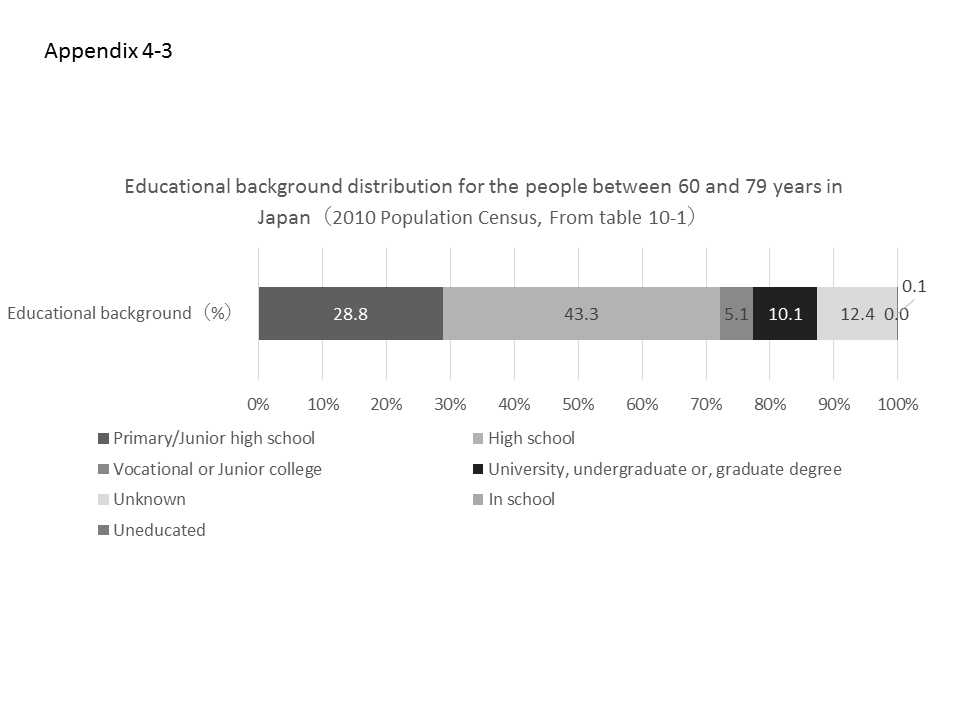

Supplement: Supplementary file 41 — Appendix 4-3 [file 10038_2018_494_MOESM41_ESM.tif]
