# Supplementary material for: A comparison of genome cohort participants’ genetic knowledge and preferences to receive genetic results before and after a genetics workshop
Source: J Hum Genet. 2018 Sep 5;63(11):1139–47. doi: 10.1038/s10038-018-0494-z (PMC8075932; doi:10.1038/s10038-018-0494-z)
Supplement: Supplementary file 36 — Appendix 1-2 [file 10038_2018_494_MOESM36_ESM.docx]

Appendix 1-2: Explanations for the genetic workshop slides

(The revisions in the slides of the PowerPoint file are shown using red font)

Slide 1: Title

Slide 2: Contents of the workshop.

Slide 3-8: Explanation regarding genes, DNA, chromosomes, cells, and their relationships.

In these slides, the size relationship among human body, cells, chromosomes, genes and DNA was showed visually because based on our genetic counseling experiences, sometimes laypeople knew each word “cell”, “gene” etc. but did not have integrated knowledge.

Slide 9-11: These slides explained the relationship between genome and genes because in genome cohort research, participants do not understand the meaning of the word “Genome”, as they did not learn the word during their formal education.

Slide 12-22: Background on genetic diseases.

These slides explained basic inheritance pattern, and the difference between “genetics” and “hereditary” because both words are represented by the same word “iden” in Japanese, and people sometimes confuse both.

Slide 23-24: Explanation regarding relation between genes and environment contribution to disease occurrence.

Slide 25-28: Explanation regarding genetic testing. Analytical validity, clinical validity, and clinical utility in genetic testing were explained following the guidelines for genetic tests and diagnoses in medical practice by the Japanese association of medical sciences.

Slide 29-32: Explanation for the importance of accurately generating a family tree for obtaining correct genetic diagnosis.

Slide 33-34: Noteworthy points while dealing with genetic information. Explanation for importance of genetic information on early detection and prevention of genetic diseases.

Slide 35: Inquiry contact address.
