# Supplementary material for: A comparison of genome cohort participants’ genetic knowledge and preferences to receive genetic results before and after a genetics workshop
Source: J Hum Genet. 2018 Sep 5;63(11):1139–47. doi: 10.1038/s10038-018-0494-z (PMC8075932; doi:10.1038/s10038-018-0494-z)
Supplement: Supplementary file 37 — Appendix 2 [file 10038_2018_494_MOESM37_ESM.docx]

Appendix 2: Comparison of genetic knowledge between our study group and those of previous studies

|  |  | Before the workshop  (n = 112) | After the workshop  (n = 112) | Yamamoto *et al.*^6^: participants enrolled in cohort study  (n = 1 031) | Yamamoto *et al.* ^6^: residents of the Tohoku area (n = 2 314) | Jallinoja and Aro^12^,  (n = 1 216) ^a^ | Haga *et al.* ^13^ (n = 300)^b^ |
| --- | --- | --- | --- | --- | --- | --- | --- |
| 1. | One can see a gene with the naked eye. | 89 | 95 | 92 | 90 | 87 | 99 |
| 2. | A gene is a disease. | 78 | 93 | 89 | 84 | 87 | 98 |
| 3. | A gene is a molecule that controls hereditary characteristics. | 72 | 71 | 61 | 65 | 63 | 84 |
| 4. | Genes are inside cells. | 89 | 94 | 86 | 86 | 55 | 91 |
| 5. | A gene is a piece of DNA. | 98 | 93 | 90 | 87 | 57 | 93 |
| 6. | A gene is a cell. | 33 | 38 | 34 | 31 | 51 | 74 |
| 7. | A gene is a part of a chromosome. | 90 | 92 | 80 | 78 | 45 | 91 |
| 8. | Different body parts include different genes. | 34 | 77 | 56 | 50 | 36 | 67 |
| 9. | Genes are bigger than chromosomes. | 74 | 83 | 78 | 70 | 41 | 83 |
| 10. | The genotype is not susceptible to human intervention. | 57 | 63 | 48 | 48 | 77 | 25 |
| 11. | It has been estimated that a person has 22 000 genes^c^. | 68 | 97 | 53 | 57 | 18 | 60 |
| 12. | Healthy parents can have a child with a hereditary disease. | 84 | 91 | 81 | 75 | 85 | 97 |
| 13. | The onset of certain diseases is due to genes, environment, and lifestyle. | 84 | 91 | 80 | 78 | 88 | 98 |
| 14. | The carrier of a disease gene may be completely healthy. | 88 | 94 | 84 | 82 | 83 | 95 |
| 15. | All serious diseases are hereditary. | 87 | 90 | 89 | 84 | 83 | 98 |
| 16 | The child of a disease gene carrier is always also a carrier of the same disease gene. | 64 | 71 | 70 | 64 | 60 | 85 |
| Overall average score (%) | | 74.3 | 83.1 | 73.3 | 70.6 | 63.5 | 83.6 |
| Overall average score (full score is 16) | | 11.89 | 13.30 | - | - | - | - |

Numbers refer to the percentage of participants who answered the question correctly. Before the workshop: Our participants’ scores before receiving a basic genetics knowledge workshop; after the workshop: Our participants’ scores after receiving a basic genetics knowledge workshop.

a; The study population in Jallinoja and Aro (1999) comprised 1 216 participants randomly selected from the general population in Finland.

The age composition of the participants was: 16–24 years, 11%; 25–44 years, 48%; 45–64 years, 41%.

b; The study population for Haga *et al*. (2013) comprised 300 participants enrolled in a type 2 diabetes mellitus genetic testing study of the general public in Durham, North Carolina, USA. The age composition of the participants was: 18-29 years, 44%; 30–39 years, 19%; 40–49 years, 16%; 50–59 years, 11%; 60–69 years, 9%; over 70 years, 1%.

c; The number of genes was changed in each study to reflect current knowledge. Jallinoja and Aro (1999) listed 7 000 genes and Haga et al. (2013) listed 22 000.
