# Supplementary material for: A comparison of genome cohort participants’ genetic knowledge and preferences to receive genetic results before and after a genetics workshop
Source: J Hum Genet. 2018 Sep 5;63(11):1139–47. doi: 10.1038/s10038-018-0494-z (PMC8075932; doi:10.1038/s10038-018-0494-z)
Supplement: Supplementary file 38 — Appendix 3 [file 10038_2018_494_MOESM38_ESM.docx]

Appendix 3: Comparison of participants’ (n=112) preferences before and after the genetics workshop

|  | (After the genetics workshop) – (Before the genetics workshop) | | Before the genetics workshop | | | After the genetics workshop | | |
| --- | --- | --- | --- | --- | --- | --- | --- | --- |
| Disease category | z-score | p^a^ | First quartile | Median | Third quartile | First quartile | Median | Third quartile |
| Lifestyle diseases | -2.117 | 0.034 | 1 | 1 | 1 | 1 | 1 | 1 |
| Pharmacogenetics | -2.703 | 0.007** | 1 | 1 | 1 | 1 | 1 | 1 |
| Adult-onset clinically actionable diseases | -1.539 | 0.124 | 1 | 1 | 1 | 1 | 1 | 1 |
| Adult-onset non-clinically actionable diseases | -2.993 | 0.003** | 1 | 1 | 1 | 1 | 1 | 2 |
| Non-clinically actionable multifactorial diseases | -3.351 | 0.001** | 1 | 1 | 1 | 1 | 1 | 2 |
| All genetic information | -3.371 | 0.001** | 1 | 1 | 2 | 1 | 2 | 2 |

a: P values were calculated using the Wilcoxon signed-rank test

**: p < 0.01, *: p < 0.05
